# Exopolysaccharides from vaginal lactobacilli modulate microbial biofilms

**DOI:** 10.1186/s12934-023-02053-x

**Published:** 2023-03-08

**Authors:** Barbara Giordani, Marina Naldi, Vanessa Croatti, Carola Parolin, Ülfet Erdoğan, Manuela Bartolini, Beatrice Vitali

**Affiliations:** 1 Department of Pharmacy and Biotechnology, University of Bologna, Bologna, Italy; 2 Yeditepe University, Istanbul, Turkey

**Keywords:** Exopolysaccharides, *Lactobacillus*, Postbiotics, Vaginal homeostasis, Biofilms

## Abstract

**Background:**

Exopolysaccharides (EPS) secreted by beneficial lactobacilli exert a plethora of positive activities, but little is known about their effects on biofilms of opportunistic vaginal pathogens and especially on biofilms of lactobacilli themselves. Here, the EPS produced by six vaginal lactobacilli, belonging to *Lactobacillus crispatus* (BC1, BC4, BC5) and *Lactobacillus gasseri* (BC9, BC12, BC14) species were isolated from cultural supernatants and lyophilized.

**Results:**

*Lactobacillus* EPS were chemically characterized in terms of monosaccharide composition by liquid chromatography (LC) analysis coupled to UV and mass spectrometry (MS) detection. Moreover, the ability of EPS (0.1, 0.5, 1 mg/mL) to stimulate the biofilm formation of lactobacilli and to inhibit the formation of pathogens’ biofilms was evaluated by crystal violet (CV) staining and 3-(4,5-dimethyl-2-thiazolyl)-2,5-diphenyl-2H-tetrazolium bromide (MTT) assay.

Isolated EPS (yields 133–426 mg/L) were heteropolysaccharides mainly composed of d-mannose (40–52%) and d-glucose (11–30%). For the first time we demonstrated that *Lactobacillus* EPS were able to stimulate in a dose-dependent manner (*p* < 0.05) the formation of biofilms of ten strains belonging to *L. crispatus*, *L. gasseri* and *Limosilactobacillus vaginali*s species, in terms of cell viability (84–282% increase at 1 mg/mL) and especially biofilm biomass (40–195% increase at 1 mg/mL), quantified with MTT assay and CV staining, respectively. EPS released from *L. crispatus* and *L. gasseri* were found to better stimulate the biofilms of the same producer species rather than that of other species, including producing strains themselves and other strains. Conversely, the biofilm formation of bacterial (*Escherichia coli*, *Staphylococcus* spp., *Enterococcus* spp. and *Streptococcus agalactiae*) and fungal (*Candida* spp.) pathogens was inhibited. The anti-biofilm activity was dose-dependent and was more marked for *L. gasseri*-derived EPS (inhibition up to 86%, 70%, and 58% at 1 mg/mL, 0.5 mg/mL, and 0.1 mg/mL, respectively)*,* whilst *L. crispatus*-derived EPS resulted overall less efficient (inhibition up to 58% at 1 mg/mL and 40% at 0.5 mg/mL) (*p* < 0.05).

**Conclusions:**

Lactobacilli-derived EPS favour the biofilm formation of lactobacilli preventing, at the same time, that of opportunistic pathogens. These results support the possible employment of EPS as postbiotics in medicine as a therapeutic/preventive strategy to counteract vaginal infections.

**Supplementary Information:**

The online version contains supplementary material available at 10.1186/s12934-023-02053-x.

## Background

Exopolysaccharides (EPS) are high molecular-weight biopolymers produced by various microorganisms during their metabolism and consist of repeating units of one (homopolysaccharides) or more (heteropolysaccharides) monosaccharides with (or without) non-carbohydrate substituents [1]. EPS can be either closely attached to bacterial surfaces, loosely associated with the cell envelope, or secreted in the surrounding environment [2, 3]. Bacterial EPS have different physiological functions in the mediation of cell recognition, in bacterial adhesion and communication, as protective agents against extracellular stresses and are a robust structural component of biofilm extracellular matrix [4, 5]. EPS are nowadays exploited in dairy, cosmetic, and pharmaceutical industries due to peculiar physico-chemical and biological features [6].

*Lactobacillus*, and related genera, are predominant in the healthy vaginal microbiota, acting as a primary defense against infections [7]. These beneficial effects have been ascribed, at least in part, to the production of bioactive metabolites (i.e. lactic acid, hydrogen peroxide, biosurfactants) [8–10]. Recently, EPS produced by lactobacilli have shown some health-promoting properties in terms of virucidal effects [11] and antimicrobial activity towards food borne pathogens [12, 13]. Moreover, lactobacilli-derived EPS bring benefits on the host through hypocholesterolemic, antihypertensive, immunomodulatory, and antioxidant activities [14–16]. Regarding the vaginal microbiota, EPS from lactobacilli revealed anti-inflammatory activities and antitumoral effects through the induction of apoptosis in cervical cancer cells [17].

EPS from lactobacilli were also found to modulate bacterial attachment and auto-aggregation mechanisms, mainly exerting inhibitory activity against bacterial pathogens biofilms [3, 18–21]. Biofilms are complex structures made of a consortium of microorganisms embedded in an extracellular matrix that ensure protection, permanence of cells to surfaces, and cell–cell interactions [22]. Extracellular matrix is a tridimensional network composed of various external polymeric substances including proteins, nucleic acids, lipids, and polysaccharides such as EPS. Pathogenic biofilms increase cell tolerance to environmental stress, conventional therapies, and host immunity, and are correlated to chronic and recurrent infections [18]. Contrariwise, the capacity of lactobacilli to form biofilms in vivo represents a protective factor allowing stable colonization of vaginal niche and providing a physical barrier against the invasion and over-proliferation of pathogenic species [23]. In this perspective, EPS could be exploited as a possible treatment (and/or prevention) of infections caused by biofilm-forming microorganisms as a new biodegradable, nontoxic, bio-compatible approach based on molecules produced by beneficial lactobacilli [5].

Despite EPS can mediate part of the beneficial effects of lactobacilli, little is known about their direct effects on the biofilms of vaginal lactobacilli.

In the present study, the EPS released by six vaginal lactobacilli, belonging to *Lactobacillus crispatus* (BC1, BC4, and BC5) and *Lactobacillus gasseri* (BC9, BC12, and BC14) species, were characterized in terms of monosaccharide composition and their effects on the biofilm of different microbial species. In particular, we first focused on the capability of EPS to stimulate the biofilm formation of lactobacilli of vaginal origin (belonging to *L. crispatus, L. gasseri* and *Limosilactobacillus vaginalis* species) with proven probiotic features. Secondly, lactobacilli-derived EPS were sought for anti-biofilm activity against a panel of bacteria and fungi responsible for vaginal dysbiosis and infections. Biofilm formation of both beneficial lactobacilli and pathogens was assessed by combining two methodologies, namely crystal violet (CV) staining and MTT assay, to gain information about total biomass and cell viability in biofilms, respectively.

## Results

### Vaginal lactobacilli produce exopolysaccharides

EPS from six lactobacilli of vaginal origin, belonging to *L. crispatus* (BC1, BC4, and BC5) and *L. gasseri* (BC9, BC12, and BC14) species, were recovered from the supernatants of stationary phase cultures and lyophilized. All lactobacilli were able to produce EPS (EPS-BC1, EPS-BC4, EPS-BC5, EPS-BC9, EPS-BC12, and EPS-BC14) with variable yields depending on the strain. Specifically, *L. gasseri* BC9 yielded the highest amount of EPS (426 ± 15 mg/L of culture) followed by *L. crispatus* BC4 (358 ± 16 mg/L of culture), while *L. crispatus* BC5 was the least productive strain (133 ± 13 mg/L of culture). The yields of EPS obtained from *L. crispatus* BC1, *L. gasseri* BC12, and *L. gasseri* BC14 were 289 ± 18 mg/L, 259 ± 9 mg/L, and 330 ± 3 mg/L of culture, respectively.

Since previous thermo gravimetric analysis (TGA) showed that lyophilization may not allow to get completely anhydrous samples [24], the moisture content was determined and resulted to be 38% in EPS-BC1, 53% in EPS-BC4, 42% in EPS-BC5, 40% in EPS-BC9, 36% in EPS-BC12, and 40% in EPS-BC14. Since, upon drying, samples are prone to re-hydrate, in all investigations carried out within this study, EPS extracts were used as hydrated.

### Exopolysaccharides from *Lactobacillus* are heteropolysaccharides

The compositions of lyophilized EPS were determined by LC-UV and LC–MS analysis. Slightly modifying a previously developed approach [25], monosaccharides released from EPS by acidic hydrolysis were derivatized with 1-phenyl-3-methyl-5-pyrazolone (PMP) and analysed by a reverse phase chromatographic approach, which provided the identification and the quantitation of 8 monosaccharides (d-mannose, d-glucosamine, d-rhamnose, d-galactosamine, d-glucose, d-galactose, d-xylose and d-fucose). An exemplificative chromatogram of PMP-monosaccharides is reported in Additional file 1: Fig. S1A, while a representative chromatogram of PMP-monosaccharide standards is depicted in Additional file 1: Fig. S1B.

The PMP-monosaccharides identification was achieved by comparing their retention time with those of standard PMP-monosaccharides (Additional file 1: Table S1) and further confirmed by the MS analysis carried out with a high-resolution quadrupole–time-of-flight hybrid mass spectrometer equipped with an electrospray ionization source (ESI-Q-TOF). The MS analysis allowed the identification of the PMP-monosaccharides based on their molecular mass, fragment ions, and retention time. An exemplificative chromatogram is reported in Additional file 1: Fig. S2.

Concerning the quantitative analysis, the range of linearity, the regression analysis, and the quantification limit for each monosaccharide are reported in Additional file 1: Table S1.

**Fig. 1:**
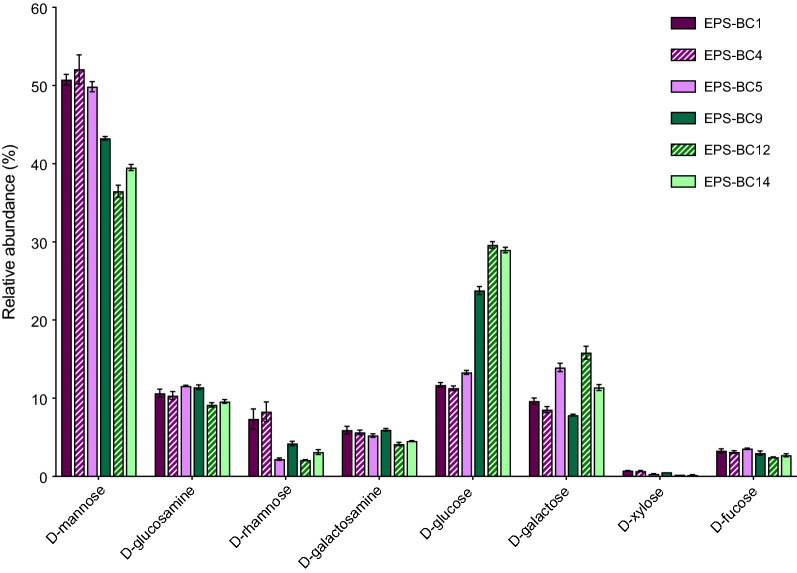
Relative abundance of monosaccharides in EPS recovered from lactobacilli. Values are the mean of analyses performed in duplicate on three independent samples.

The monosaccharide composition of EPS is visualized in Fig. 1 that reports the relative abundance of each monosaccharide. Notwithstanding differences in the relative abundance of monosaccharides within the same species were encountered, d-mannose was the most abundant monosaccharide in EPS from all lactobacilli (39–52%), followed by d-glucose (11–30%) and d-galactose (8–16%), whereas the relative abundance of d-xylose and d-fucose were very low (< 1% and 3–4%, respectively).

**Fig. 2:**
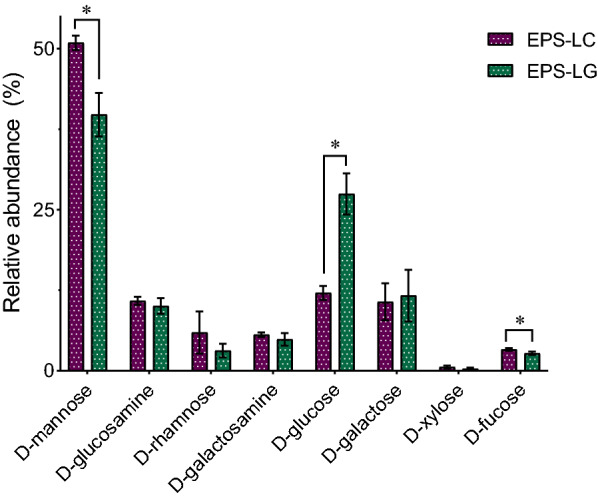
Average relative abundance of monosaccharides in EPS recovered from *L. crispatus* (EPS-LC) and *L. gasseri* (EPS-LG) strains (mean ± SD, *n* = 3). * *p* < 0.05 (Student’s t-test)

To better highlight differences between EPS from the two *Lactobacillus* species, EPS were grouped according to the producing species (Fig. 2). A comparison of the EPS composition from the two *Lactobacillus* species revealed significant differences in d-mannose, d-glucose and d-fucose. In detail, the relative abundances of d-mannose and d-fucose resulted significantly higher in EPS from *L. crispatus* (EPS-LC) strains than in EPS from *L. gasseri* (EPS-LG) strains, while the relative abundance of d-glucose was higher in EPS-LG (*p* < 0.05, Student’s t-test) (Fig. 2).

### Exopolysaccharides stimulate biofilms of lactobacilli

EPS are components of extracellular matrix of microbial biofilms, including those of lactobacilli [5]. Therefore, EPS recovered from *L. crispatus* (BC1, BC4, and BC5) and *L. gasseri* (BC9, BC12, and BC14) were initially sought for their effects on the formation of biofilms of 10 lactobacilli strains of vaginal origin. The panel of lactobacilli employed for testing included the EPS-producing strains themselves (i.e. BC1, BC4, BC5, BC9, BC12, and BC14) and other strains belonging to the species *L. crispatus* (BC3), *L. gasseri* (BC10) and *L. vaginalis* (BC16 and BC17). EPS were tested at three concentrations (0.1, 0.5, and 1 mg/mL) and formation of lactobacilli biofilms was assessed by two methods, namely crystal violet (CV) staining and MTT assay, which provide diverse and complementary information. Specifically, CV quantifies the total biofilm biomass since it is a non-specific dye which binds cellular components of live and dead cells, as well as the extracellular matrix. Instead, MTT is enzymatically reduced and converted into a formazan salt only by metabolically active cells, thus allowing the quantification of viable cells in biofilms.

**Fig. 3:**
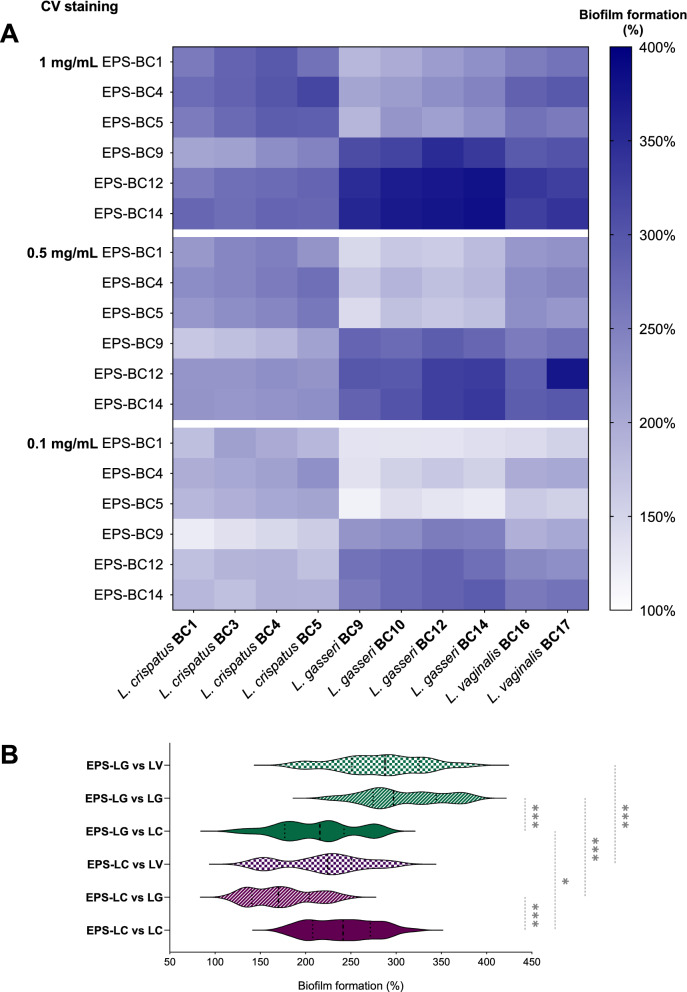
Effects of *Lactobacillus* EPS on the biofilms of lactobacilli assessed by CV staining. **A** Heatmap of the activity of EPS-BC1, EPS-BC4, EPS-BC5, EPS-BC9, EPS-BC12, and EPS-BC14 at three concentrations (0.1, 0.5, and 1 mg/mL) on the formation of biofilms of *L. crispatus* (BC1, BC3, BC4, BC5), *L. gasseri* (BC9, BC10, BC12, BC14) and *L. vaginalis* (BC16, BC17). Results are reported in percentage with respect to control (100%) (*n* = 3). **B** Violin plot of the overall activity of EPS-LC and EPS-LG *versus* biofilms of lactobacilli grouped by species (*L. crispatus*, LC; *L. gasseri*, LG; *L. vaginalis*, LV). Solid and dotted lines represent median and quartiles, respectively. * *p* < 0.033; *** *p* < 0.001 (ANOVA)

**Fig. 4:**
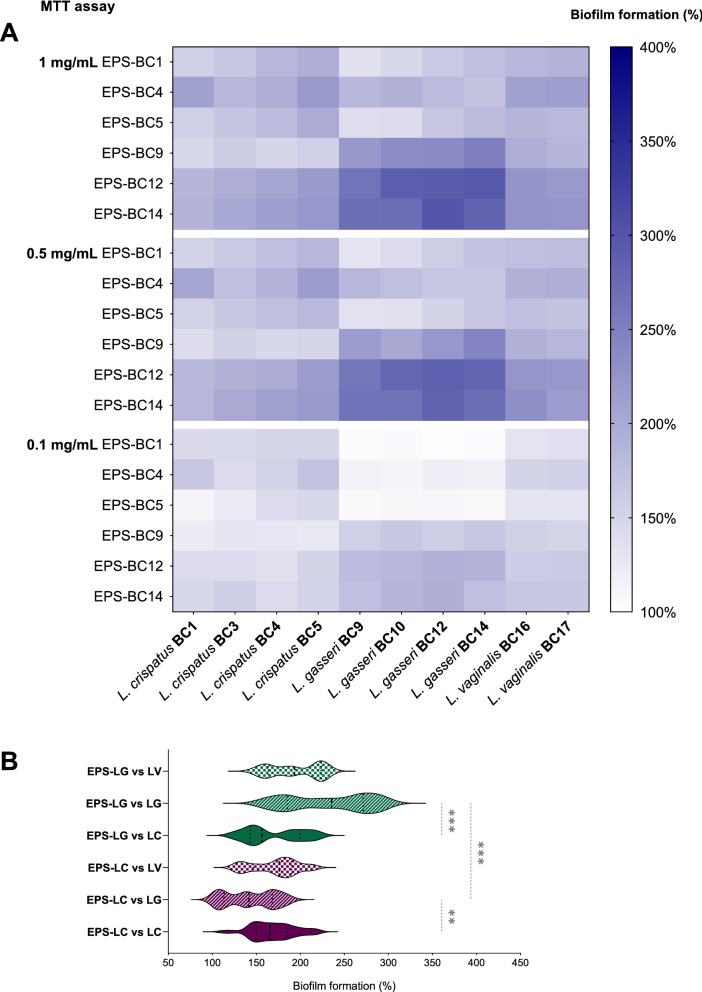
Effects of *Lactobacillus* EPS on the biofilms of lactobacilli assessed by MTT assay. **A** Heatmap of the activity of EPS-BC1, EPS-BC4, EPS-BC5, EPS-BC9, EPS-BC12, and EPS-BC14 at three concentrations (0.1, 0.5, and 1 mg/mL) on the formation of biofilms of *L. crispatus* (BC1, BC3, BC4, BC5), *L. gasseri* (BC9, BC10, BC12, BC14) and *L. vaginalis* (BC16, BC17). Results are reported in percentage with respect to control (100%) (*n* = 3). **B** Violin plot of the overall activity of EPS-LC and EPS-LG *versus* biofilms of lactobacilli grouped by species (*L. crispatus*, LC; *L. gasseri*, LG; *L. vaginalis*, LV). Solid and dotted lines represent median and quartiles, respectively. ** *p* < 0.002; *** *p* < 0.001 (ANOVA)

Results are reported in percentages of biofilm formation compared to controls, i.e. biofilm formed in the absence of EPS (considered as 100%), and summarized as heatmaps in Fig. 3A (CV staining) and Fig. 4A (MTT assay). Results are also more extensively represented as histograms in Additional file 1: Fig. S3 (CV staining) and Additional file 1: Fig. S4 (MTT assay). The data clearly revealed that the presence of EPS strongly increased the formation of biofilm biomass of all lactobacilli considered (*p* < 0.05, ANOVA) (Fig. 3A, Additional file 1: Fig. S3). The viability of lactobacilli was also enhanced when biofilms were established in the presence of EPS (*p* < 0.05, ANOVA) (Figs. 4A, Additional file 1: Fig. S4).

Furthermore, CV staining and MTT assay highlighted that the effects of EPS on biofilms were dose-dependent, as the stimulation of lactobacilli biofilms significantly differed at the three tested concentrations (*p* < 0.05, ANOVA). In particular, the promotion of biofilm formation was higher in the presence of EPS 1 mg/mL, which allowed for an average formation of lactobacilli biofilm biomass ranging from 244% (EPS-BC1) to 326% (EPS-BC14) (CV staining), as well as an average lactobacilli viability in biofilms ranging from 169% (EPS-BC1) to 240% (EPS-BC14) (MTT assay). Halving the concentration of tested EPS (0.5 mg/mL), the average formation of lactobacilli biofilm biomass was between 204% (EPS-BC1) and 281% (EPS-BC12) (CV staining), whilst the average lactobacilli viability in biofilms was between 160% (EPS-BC5) and 235% (EPS-BC14) (MTT assay). Even at the lowest concentration considered, EPS significantly stimulated the biofilms of all lactobacilli (*p* < 0.05, ANOVA), with an average lactobacilli biofilm biomass formation between 160% (EPS-BC1) and 237% (EPS-BC14) (CV staining) and an average lactobacilli viability in biofilms between 121% (EPS-BC5) and 165% (EPS-BC14). Taken together, the data collected indicate that the average biofilm formation was 281% (CV staining)/201% (MTT assay), 237% (CV staining)/194% (MTT assay) and 194% (CV staining)/144% (MTT assay) at EPS concentrations of 1 mg/mL, 0.5 mg/mL, and 0.1 mg/mL, respectively.

To evaluate the agreement between the two methods, a correlation analysis between datasets collected through CV staining and MTT assay was conducted (Additional file 1: Fig. S7A). The Person’s correlation coefficient was found to be 0.8855, indicating a good correlation between the two methods employed for biofilm quantification. However, the percentages of biofilm formation detected by CV staining were significantly higher than those obtained by MTT assay (*p* < 0.05, Wilcoxon paired-rank test). This finding indicates that the addition of EPS to lactobacilli cultures promoted a massive formation of biofilm biomass and extracellular matrix and, to a lesser extent, the embedding of metabolically active cells in the biofilms.

Data reported in Figs. 3, 4A were then grouped according to the EPS-producing species (EPS-LC and EPS-LG) to sought for possible differences in stimulation capacity between EPS produced by *L. crispatus* and *L. gasseri*. Moreover, to investigate whether a *Lactobacillus* species was more affected by EPS-LC or EPS-LG, results on biofilm formation were also grouped by single lactobacilli species i.e., *L. crispatus* (LC), *L. gasseri* (LG), and *L. vaginalis* (LV). The results are depicted in violin plots in Fig. 3B (CV staining) and Fig. 4B (MTT assay), showing the frequency distribution of the data of EPS-LC/EPS-LG at all concentrations *versus* LC, LG, and LV biofilms. It is worth noting that, overall, EPS produced by a *Lactobacillus* species (LC or LG) stimulated more the formation of biofilms of the same species rather than that of lactobacilli belonging to other species. Indeed, EPS-LC promoted the formation of LC biofilm biomass (median biofilm formation of 241%) significantly more than that of LG (170%; *p* < 0.05, ANOVA) and slightly more than that of LV (225%) (CV staining, Fig. 3B). EPS-LC also enhanced the viability of LC in biofilms (median biofilm formation of 176%) significantly more than that of LG (142%; *p* < 0.05, ANOVA) and slightly more than that of LV (166%) (MTT assay, Fig. 4B).

Among EPS-LC, EPS-BC4 resulted more effective in stimulating the formation of LC biofilms biomass (average of 251%, CV staining) and LC viability in biofilms (average of 185%, MTT assay) than EPS-BC1 and EPS-BC5 (*p* < 0.05, ANOVA). No significant differences were found between EPS-BC1 (234%, CV staining; 163%, MTT assay) and EPS-BC5 (237%, CV staining; 158%, MTT assay).

EPS-LG stimulated the formation of LG biofilm biomass (median biofilm formation of 297%) significantly more than that of LC (216%; *p* < 0.05, ANOVA) and slightly more than that of LV (288%) (CV staining, Fig. 3B). In addition, EPS-LG increased the viability of LG in biofilms (median biofilm formation of 236%) significantly more than that of LC and LV (156% and 194%, respectively; *p* < 0.05, ANOVA) (MTT assay, Fig. 4B).

EPS-BC12 and EPS-BC14 showed similar behaviour towards LG biofilms, both in terms of biofilm biomass formation (average of 317–319%, CV staining) and viability of LG in biofilms (average of 245–249%, MTT assay), and were more effective than EPS-BC9 (283%, CV staining; 206%, MTT assay) (*p* < 0.05, ANOVA).

Interestingly, all EPS from *L. crispatus* or *L. gasseri* strains similarly favoured the biofilm formation of lactobacilli strains belonging to the same species (LC or LG), including producing strains themselves and other strains (*p* > 0.05, ANOVA).

Furthermore, the formation of biofilm biomass of a *Lactobacillus* species (LC or LG) resulted more stimulated by EPS produced by the same species (EPS-LC or EPS-LG) than by EPS from the other species (CV staining, Fig. 3B). Considering the MTT assay, the viability of LG in biofilms was markedly higher in the presence of EPS-LG (median biofilm formation of 297%) than in the presence of EPS-LC (170%) (*p* < 0.05, ANOVA). The viability of LC in biofilms was instead slightly higher in the presence of EPS-LC (median biofilm formation of 176%) than in the presence of EPS-LG (156%) (MTT assay, Fig. 4B). The results towards LV biofilms were less clear-cut, since this species resulted more affected by EPS-LC for what concern biofilm biomass formation (CV staining, Fig. 3B), while its viability was more stimulated by EPS-LG (MTT assay, Fig. 4B).

### Exopolysaccharides impair biofilms of opportunistic pathogens

To exclude undesired stimulating effects and to investigate the potential anti-biofilm activity, EPS from *L. crispatus* (BC1, BC4, and BC5) and *L. gasseri* (BC9, BC12, and BC14) were assessed towards biofilms of vaginal opportunistic pathogens. These included Gram-negative bacteria (*Escherichia coli* DSM1900 and *E. coli* DSM18039), Gram-positive bacteria (*Staphylococcus aureus* DSM2569, *S. aureus* SO88, *Staphylococcus lugdunensis* BC102, *Enterococcus faecalis* BC101, *Enterococcus faecium* BC105, and *Streptococcus agalactiae* SO104) and fungi (*Candida albicans* SO1 and *Candida glabrata* SO17). Following the same approach as for lactobacilli biofilms, EPS were tested at three concentrations (0.1, 0.5, and 1 mg/mL) and biofilm formation was quantified by CV staining and MTT assay.

**Fig. 5:**
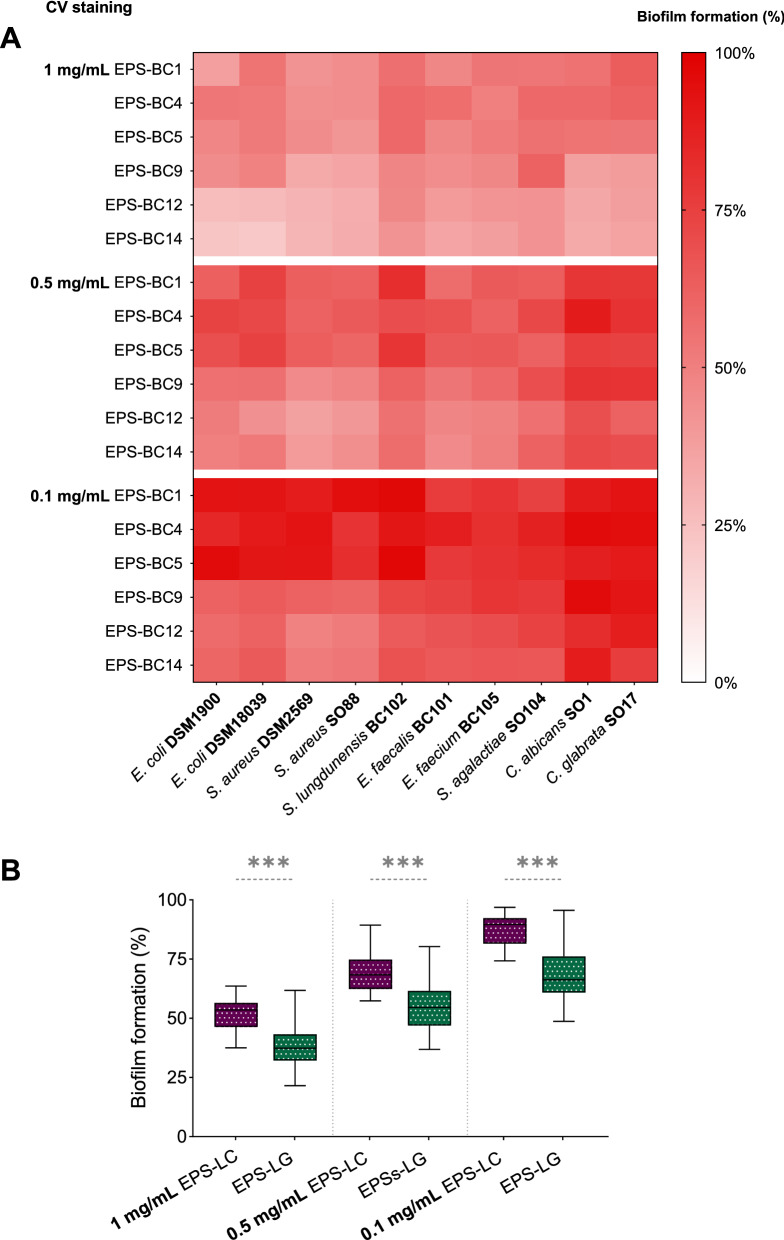
Effects of *Lactobacillus* EPS on the biofilms of pathogens assessed by CV staining. **A** Heatmap of the activity of EPS-BC1, EPS-BC4, EPS-BC5, EPS-BC9, EPS-BC12, and EPS-BC14 at three concentrations (0.1, 0.5, and 1 mg/mL) on the formation of biofilms of *E. coli* (DSM1900, DSM18039), *S. aureus* (DSM2569, SO88), *S. lugdunensis* BC102, *E. faecalis* BC101, *E. faecium* BC105, *S. agalactiae* SO104, *C. albicans* SO1 and *C. glabrata* SO17. Results are reported in percentage with respect to control (100%) (*n* = 3). **B** Boxplot of the anti-biofilm activity exerted by EPS-LC and EPS-LG at three concentrations. Each box represents the interquartile range (25–75th percentile). Lines within the boxes indicate the median values of the samples. The extremes of the bars indicate the minimum and the maximum value, respectively. *** *p* < 0.001 (ANOVA)

**Fig. 6:**
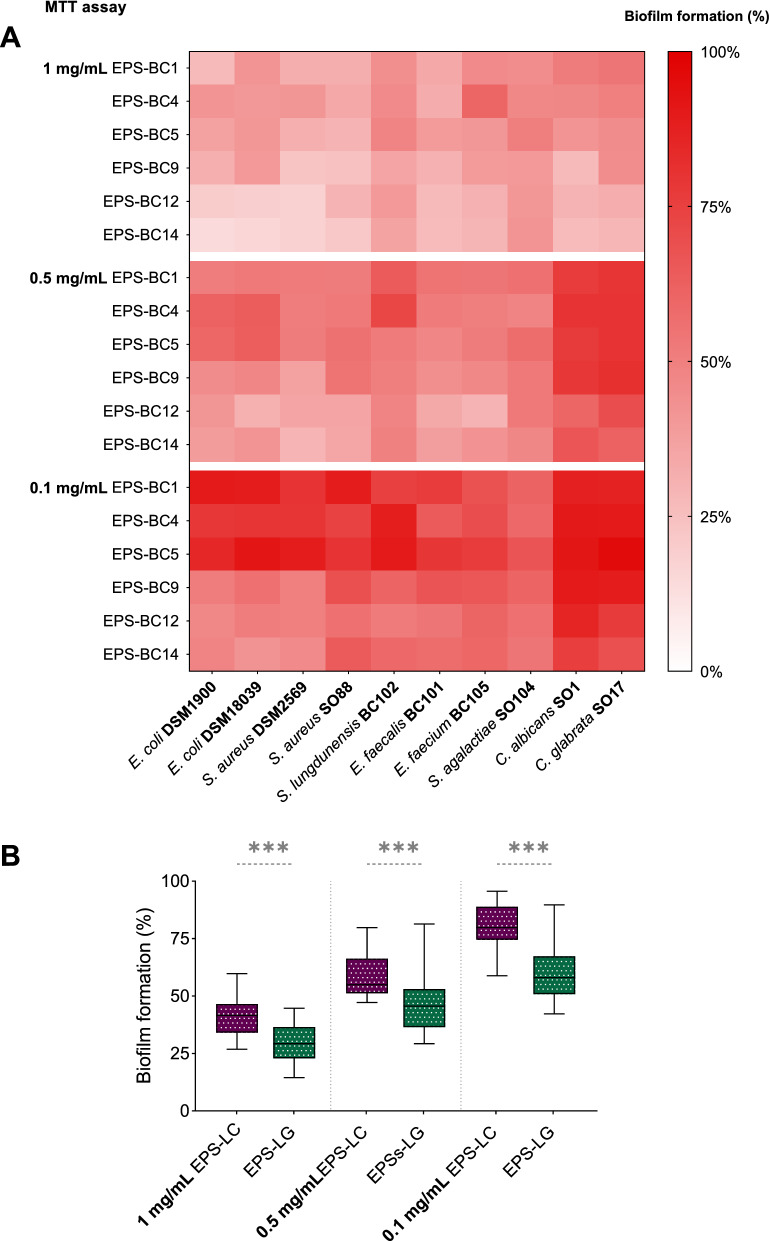
Effects of *Lactobacillus* EPS on the biofilms of pathogens assessed by MTT assay. **A** Heatmap of the activity of EPS-BC1, EPS-BC4, EPS-BC5, EPS-BC9, EPS-BC12, and EPS-BC14 at three concentrations (0.1, 0.5, and 1 mg/mL) on the formation of biofilms of *E. coli* (DSM1900, DSM18039), *S. aureus* (DSM2569, SO88), *S. lugdunensis* BC102, *E. faecalis* BC101, *E. faecium* BC105, *S. agalactiae* SO104, *C. albicans* SO1 and *C. glabrata* SO17. Results are reported in percentage with respect to control (100%) (*n* = 3). **B** Boxplot of the anti-biofilm activity exerted by EPS-LC and EPS-LG at three concentrations. Each box represents the interquartile range (25–75th percentile). Lines within the boxes indicate the median values of the samples. The extremes of the bars indicate the minimum and the maximum value, respectively. *** *p* < 0.001 (ANOVA)

The percentages of biofilm formation compared to controls without EPS (100%) were determined and results are summarized as heatmaps in Fig. 5A (CV staining) and Fig. 6A (MTT assay). Results are also more extensively represented as histograms in Additional file 1: Fig. S5 (CV staining) and Additional file 1: Fig. S6 (MTT assay).

Importantly, incubation with EPS reduced both the formation of total biofilm biomass (CV staining, Figs. 5A and Additional file 1: Fig. S5) and the viability in biofilms (MTT assay, Figs. 6A and Additional file 1: Fig. S6) of all pathogens tested. The anti-biofilm effect was dose-dependent and significantly decreased when EPS concentration was lowered (*p* < 0.05, ANOVA). Considering the data collected from all EPS treatments, the average formation of pathogens biofilms was 45% (CV staining)/35% (MTT assay), 62% (CV staining)/54% (MTT assay) and 78% (CV staining)/71% (MTT assay) at EPS concentrations of 1 mg/mL, 0.5 mg/mL, and 0.1 mg/mL, respectively. More in detail, 1 mg/mL EPS significantly reduced the biomass of pathogens biofilms by 46% (EPS-BC4) to 67% (EPS-BC14) (CV staining), and the viability by 56% (EPS-BC4) to 74% (EPS-BC14) (MTT assay) (*p* < 0.05, ANOVA). The intermediate EPS concentration still significantly impaired the development of biofilm biomass (reduction of 29–49%) and the viability of pathogens in biofilms (reduction of 39–56%), with the only exception of EPS-BC4 towards *C. albicans* SO1 (CV staining, Additional file 1: Fig. S5B). At the lowest tested concentration (0.1 mg/mL EPS), the anti-biofilm activity was mostly lost, and inhibition ability varied depending on the EPS and pathogen considered. At this concentration, the best anti-biofilm profiles were observed for EPS-BC9, EPS-BC12, and EPS-BC14 (Additional file 1: Fig. S5, S6D–E), which were able to significantly reduce the biofilms of all bacterial strains with inhibition percentages of 20–40% (CV staining)/32–50% (MTT assay), 26–51% (CV staining)/40–54% (MTT assay) and 32–49% (CV staining)/36–58% (MTT assay), respectively. EPS-BC12 and EPS-BC14 at a concentration of 0.1 mg/mL also significantly hampered the biofilm formation of *C. glabrata* SO17 (inhibition: 11–24%, CV staining; 23–32%, MTT assay) and viability of *C. albicans* SO1 in biofilm (inhibition: 14–25%, MTT assay), but had no impact on the biofilm biomass formation of the latter (CV assay). *C. albicans* SO1 and *C. glabrata* SO17 biofilms were not affected by 0.1 mg/mL EPS-BC1, EPS-BC4, EPS-BC5, and EPS-BC9, thus suggesting that high concentrations of EPS are required to effectively counteract *Candida* spp. biofilms.

Not all bacterial biofilms were reduced by 0.1 mg/mL EPS-BC1, EPS-BC4, and EPS-BC5 (Additional file 1: Figs. S5, S6A–C). In general, these EPS seemed to be more effective against biofilms of *E. faecium* BC105 and *S. agalactiae* SO104, which were significantly reduced by 19–20% (CV staining)/24–32% (MTT assay) and 13–26% (CV staining)/33–41% (MTT assay), respectively. EPS-BC1 and EPS-BC5 also significantly affected the formation of biofilm of *E. faecalis* BC101, whilst biofilm of *S. aureus* SO88 was reduced by18-20% (CV staining)/20–26% (MTT assay) with EPS-BC4 and EPS-BC5.

Data reported in Figs. 5, 6A were also grouped according to the EPS-producing species (EPS-LC and EPS-LG) and visualized in box plots in Fig. 5B (CV staining) and Fig. 6B (MTT assay), clearly showing that anti-biofilm activity exerted by EPS-LG was significantly higher than that of EPS-LC at all tested concentrations. As described above, these differences were particularly evident when EPS were tested at 0.1 mg/mL.

Furthermore, EPS-BC12 and EPS-BC14 proved to be more efficient in counteracting bacterial and fungal pathogens biofilms than the other EPS tested (considering all concentrations towards all pathogens and CV staining/MTT assay; *p* < 0.05, Wilcoxon paired-rank test), followed by EPS-BC9. Instead, no significant difference in anti-biofilm activity was found between EPS-BC1, EPS-BC4, and EPS-BC5.

Finally, the correlation analysis between datasets collected by CV staining and MTT assay (Additional file 1: Fig. S7B) revealed a Person’s correlation coefficient of 0.9397, indicating a strong correlation between the two methods employed for the quantification of pathogens biofilms. The percentages of pathogens biofilm formation obtained with CV staining were only slightly lower than those obtained MTT assay (*p* > 0.05, Wilcoxon paired-rank test), suggesting that EPS were very effective in reducing not only pathogens viability inside biofilms, but also the formation of a wide biofilm structure.

### Effects of exopolysaccharides on mixed biofilms

**Fig. 7:**
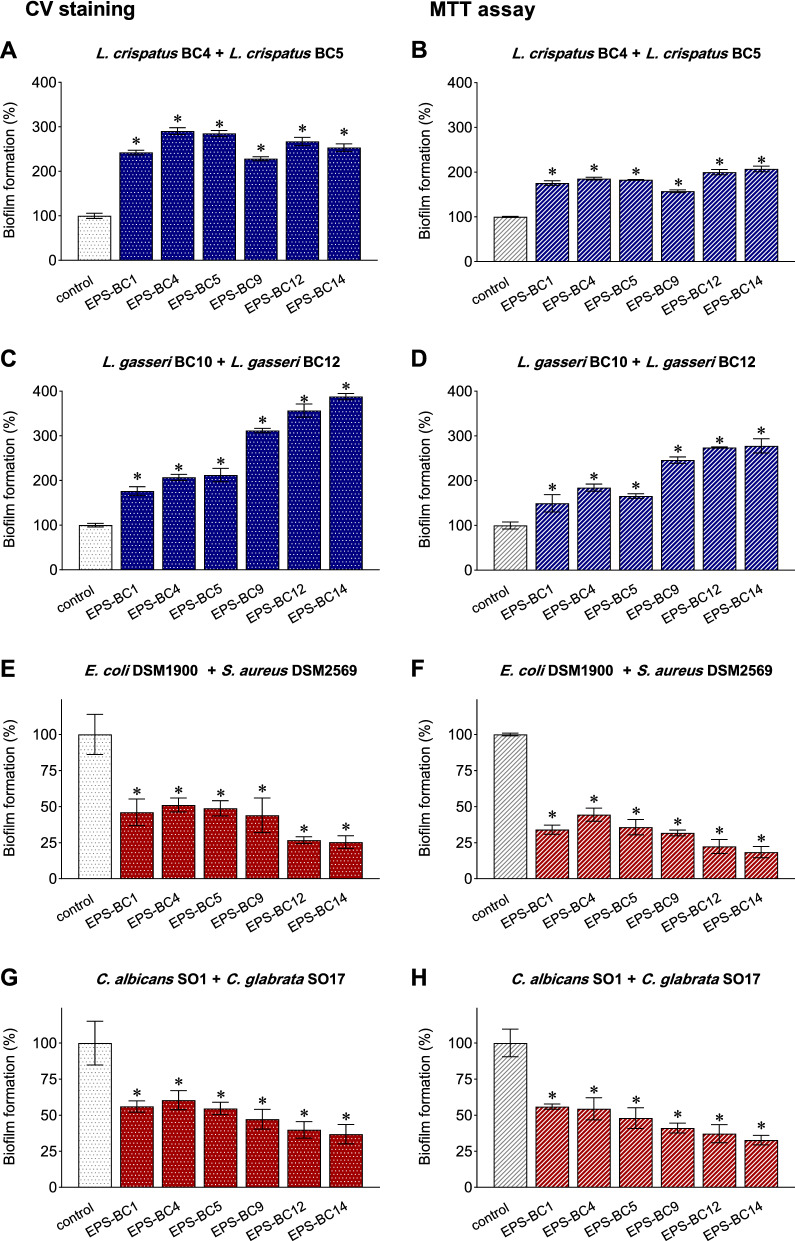
Effects of *Lactobacillus* EPS (1 mg/mL) on mixed biofilms: (**A**) *L. crispatus* BC4 + *L. crispatus* BC5, assessed by CV staining; (**B**) *L. crispatus* BC4 + *L. crispatus* BC5, assessed by MTT assay; (**C**) *L. gasseri* BC10 + *L. gasseri* BC12, assessed by CV staining; (**D**) *L. gasseri* BC10 + *L. gasseri* BC12, assessed by MTT assay; (**E**) *E. coli* DSM1900 + *S. aureus* DSM2569, assessed by CV staining; (**F**) *E. coli* DSM1900 + *S. aureus* DSM2569, assessed by MTT assay; (**G**) *C. albicans* SO1 + *C. glabrata* SO17, assessed by CV staining; (**H**) *C. albicans* SO1 + *C. glabrata* SO17, assessed by MTT assay. The results are expressed in percentage with respect to control (100%) (mean ± SD, n = 3). * *p* < 0.05 (ANOVA)

To better reflect the complexity of the physiological environment, the effects of EPS from *L. crispatus* (BC1, BC4, and BC5) and *L. gasseri* (BC9, BC12, and BC14) were further evaluated towards mixed biofilms of lactobacilli, obtained by co-culturing two *L. crispatus* strains (BC4 and BC5) and two *L. gasseri* strains (BC10 and BC12). EPS were tested at 1 mg/mL, which resulted the most effective concentration in stimulating the formation of *Lactobacillus* biofilms. The formation of biofilms was assessed through CV staining and MTT assay and results are depicted in Fig. 7A–D.

The data highlighted that all EPS tested strongly improved mixed biofilms of lactobacilli. Indeed, both the formation of biofilm biomass and the viability of lactobacilli in the biofilms were significantly enhanced in the presence of EPS with respect to the control (*p* < 0.05, ANOVA). In particular, *L. crispatus* BC4 + *L. crispatus* BC5 biofilm biomass ranged from 228% (EPS-BC9) and 291% (EPS-BC4) (Fig. 7A, CV staining), while their viability in biofilms varied between 157% (EPS-BC9) and 207% (EPS-BC14) (Fig. 7B, MTT assay); *L. gasseri* BC10 + *L. gasseri* BC12 biofilm biomass ranged from 177% (EPS-BC1) and 388% (EPS-BC14) (Fig. 7C, CV staining), while their viability in biofilms was between 150% (EPS-BC1) and 278% (EPS-BC14) (Fig. 7D, MTT assay).

The results obtained for *L. crispatus* BC4 + *L. crispatus* BC5 and *L. gasseri* BC10 + *L. gasseri* BC12 mixed biofilms were very similar to those found for *L. crispatus* (BC4 or BC5) biofilms and for *L. gasseri* (BC10 or BC12 biofilms), respectively (Additional file 1: Figs. S3, S4) (*p* > 0.05, ANOVA), thus demonstrating that EPS maintained the stimulating capability over multistrains biofilms.

Moreover, the effect of 1 mg/mL EPS was assessed against mixed biofilms of opportunistic pathogens, namely aerobic bacteria (*E. coli* DSM1900 co-cultured with *S. aureus* DSM2569) and fungi (*C. albicans* SO1 co-cultured with *C. glabrata* SO17). Results from CV staining and MTT assay are reported in Fig. 7E–H and revealed that *Lactobacillus* EPS efficiently hampered mixed biofilms of pathogens, confirming the anti-biofilm potential towards vaginal harmful microorganisms. Specifically, EPS inhibited *E. coli* DSM1900 + *S. aureus* DSM2569 biofilm biomass by 49% (EPS-BC4) to 73% (EPS-BC12) (Fig. 7E) and *C. albicans* SO1 + *C. glabrata* SO17 biofilm biomass by 37% (EPS-BC4) to 63% (EPS-BC14) (Fig. 7G, CV staining). The viability of pathogens in biofilms was also greatly reduced, with inhibition percentages of 66–82% towards *E. coli* DSM1900 + *S. aureus* DSM2569 biofilm (Fig. 7F) and 44–67% against *C. albicans* SO1 + *C. glabrata* SO17 biofilm (Fig. 7H, MTT assay).

## Discussion

Lactobacilli-derived EPS have been reported to reduce pathogens biofilms and, contemporarily, to stimulate the growth of the healthy gut microflora [3, 18–22]. Besides this evidence, EPS from *Lactobacillus* spp. of vaginal origin are poorly characterized and the functionality in the vaginal microbiota is little understood [17].

Here, we recovered EPS secreted by six vaginal *Lactobacillus* strains belonging to *L. crispatus* and *L. gasseri* species, which are highly represented in the vaginal niche under eubiosis [7]. *L. crispatus* BC1, *L. crispatus* BC4, *L. crispatus* BC5, *L. gasseri* BC9*, L. gasseri* BC12, and *L. gasseri* BC14 were found to produce EPS in a variable amount, depending on the strain considered. Although many lactobacilli are not able to release high quantity of EPS (> 100 mg/mL) in cultural broth [26], *L. crispatus* and *L. gasseri* strains employed in this study can be considered good producers of EPS, in line with other studies reporting EPS yields of 170–340 mg/mL and 178–255 mg/mL for *L. crispatus* and *L. gasseri*, respectively [17, 27].

To investigate EPS sugar composition, LC-UV and LC–MS analyses were performed. All EPS resulted to be heteropolysaccharides, composed of different monosaccharides. In accordance with this finding, other authors identified lactobacilli-derived EPS as heteropolysaccharides containing two or more monosaccharides including rhamnose, arabinose, mannose, galactose, glucose, glucosamine and fucose [12, 26, 28–30]. Grouping EPS by *Lactobacillus* producer species, EPS from *L. crispatus* were found to contain more d-mannose and d-fucose than *L. gasseri*-derived EPS, which were instead characterized by a higher abundance of d-glucose. Up to now, only a few studies describe the sugar composition of EPS secreted by *L. crispatus* and *L. gasseri* species from vaginal microbiota [17, 27]. In particular, Donnarumma et al. isolated the EPS from a strain of *L. crispatus*, and identified it as homopolysaccharide composed by monomers of mannose and its derivatives [27]. Similarly, we demonstrated that d-mannose was the most abundant monomer in *L. crispatus* EPS. Other authors found out that EPS isolated from *L. gasseri* strains contained almost exclusively monomers of glucose [17], whilst *L. gasseri* strains included in our study produced heteropolysaccharides mainly composed of mannose repetitions followed by d-glucose. d-glucose content in *L. gasseri* EPS was significantly higher than that found in EPS from *L. crispatus,* indicating that distinct species of lactobacilli produce EPS with different features. Some differences in EPS structure among strains belonging to the same species are expected since EPS biosynthetic pathway is complex and can also vary depending on the environmental conditions. Indeed, monosaccharide composition can change with the growth conditions and/or the availability of sugar substrates, which affect the EPS biosynthetic pathway or the stability of the biosynthetic enzymes [26].

In addition to the sugar composition, described in the present work, other chemical features of the EPS, such as molecular weight, length and branching could affect the biological functionality [30] and are planned to be investigated in future studies.

In the present study, *Lactobacillus* EPS were functionally characterized for their impact on microbial biofilms, in terms of anti-biofilm activity towards opportunistic pathogens and, for the first time, of the stimulating effects on biofilm formation of beneficial vaginal lactobacilli.

Notably, EPS recovered from all lactobacilli were able to effectively stimulate the formation of biofilms of different strains of lactobacilli belonging to *L. crispatus*, *L. gasseri* and *L. vaginalis* species. In an attempt to simulate the in vivo complexity, mixed biofilms were also investigated. In the vaginal niche of healthy women lactobacilli can reach 80% of the whole microbial content, and a single *Lactobacillus* species is usually dominant in each woman [7]. Thus, mixed biofilms were obtained by co-culturing two strains of *L. crispatus* or two strains of *L. gasseri*. Importantly, the stimulation capability of all EPS was similar to that obtained towards single lactobacilli biofilms, indicating that EPS are able to exert an important stimulating activity even when two lactobacilli strains are simultaneously present.

In particular, results highlighted that EPS stimulation mostly affects the formation of the biofilm biomass and, to a lesser extent, the cell viability. Moreover, the effects of EPS were dose-dependent, with the highest values of biofilm biomass (average 281%) and biofilm viability (average 201%) being reached when the highest concentration of EPS tested (1 mg/mL) was supplied.

Interestingly, and generally speaking, EPS released from *L. crispatus* or *L. gasseri* species were found to better stimulate the biofilm biomass and biofilm cell viability of the same producer species rather than other species, including producing strains themselves and other strains.

To date, some studies reported the ability of EPS from the gut microbiota to modulate the colonization of beneficial species, enhancing lactobacilli and bifidobacteria growth [21, 31]. To the best of our knowledge, we here demonstrated for the first time that biofilms of lactobacilli of vaginal origin can benefit from the stimulating action of EPS recovered from lactobacilli isolated from vaginal niche.

Importantly, the biofilm-promoting activity exerted by *Lactobacillus* EPS was specific for lactobacilli, whilst biofilms of different bacterial and fungal pathogens were not stimulated.

Up to now, there is no information regarding the effects of EPS on probiotic biofilms and the molecular mechanisms beneath this activity are still to be elucidated. Nonetheless, lactobacilli-derived EPS with different chemical structures were reported to selectively increase the growth of *Lactobacillus* and *Bifidobacterium* species, thus acting as prebiotics [21, 32, 33]. Also, probiotic species can express EPS degradative extracellular enzymes belonging to glycoside hydrolase families, such as α-galactosidase and β-galactosidase, and an enzyme active toward gluco-oligosaccharides, which allows them to use EPS as carbon source [21, 34]. In this perspective, EPS from vaginal lactobacilli could support probiotics’ biofilm formation by boosting cell vitality. This is coherent with the observation that cell viability of lactobacilli in biofilms increased in the presence of EPS. However, *Lactobacillus* EPS was found to promote more the biofilm biomass than the cell viability in biofilms, thus it is likely that other mechanisms are also implicated. Some authors reported that *L. rhamnosus*-derived EPS was involved in the adhesion to intestinal cells, suggesting that EPS from lactobacilli can act as adhesins for *Lactobacillus* and related genera [35]. At the same time, since EPS are one of the main components of the biofilm extracellular matrix, their availability in the medium can enhance the structure of lactobacilli biofilms.

On the other hand, lactobacilli-derived EPS did not support the growth of non-probiotic bacteria found in the gut, such as *E. coli*, *Enterobacter aerogenes* and *Enterococcus faecalis*, hence demonstrating a specificity in the their activity [21, 32].

Concerning the vaginal ecosystem, we highlighted that *Lactobacillus* EPS effectively inhibited the formation of biofilms of microorganisms responsible for common vaginitis (*E. coli*, *Staphylococcus* spp., *Enterococcus* spp.) [36] and vaginosis (*Candida* spp.) [37], as well as severe perinatal infections (*S. agalactiae*) [38]. In particular, aerobic vaginitis and vulvovaginal candidiasis share a strong inflammatory response and a drastic reduction in the number of lactobacilli, with the concomitant over-proliferation of aerobic bacteria, mainly *E. coli* and *S. aureus*, and *Candida* spp., especially *C. albicans* and *C. glabrata*, respectively. To mimic these conditions, two mixed biofilms, one obtained co-culturing *E. coli* DSM1900 with *S. aureus* DMS2569 and the other one formed co-culturing *C. albicans* SO1 with *C. glabrata* SO17, were setup. It is worth noting that EPS solutions tested at 1 mg/mL were able to effectively counteract the formation of mixed biofilms, suggesting that they act as promising anti-biofilm agents even in complex environments, more resembling physiological situations.

Coherently to what previously observed, the anti-biofilm activity was dose-dependent, reaching the lowest values of biofilm biomass (average 45%) and biofilm viability (average 35%) with the highest concentration of EPS tested (1 mg/mL).

Moreover, at all tested concentrations, EPS recovered from *L. gasseri* resulted more active than EPS recovered from *L. crispatus* in inhibiting the biofilm formation of all pathogens. This difference was more evident at the lowest concentration of EPS (0.1 mg/mL), especially on *Candida* isolates. These data agree with those reported in the literature showing that EPS from lactobacilli demonstrated an anti-biofilm effect in a dose-dependent way towards a broad spectrum of bacterial pathogens [3, 18–21, 39, 40]. Up to now, there is no information regarding the effect of *Lactobacillus* EPS on *Candida* biofilm, rather, it is known their antimicrobial and anti-adhesive effect on some *Candida* isolates [27, 41]. Here, we reported for the first time the anti-biofilm effect of vaginal *Lactobacillus* EPS against *Candida* species, underlying the importance of EPS in preventing the insurgence of vaginal infections.

Notwithstanding continuous progress in the field, the mechanisms underpinning EPS anti-biofilm effect are still poorly understood and several hypotheses have been proposed. EPS may act as surfactants changing the physical characteristics of microbial surfaces and reducing cell-to-cell interactions, thus inhibiting the initial auto-aggregation and cell attachment to surface [20]. By transcriptomic analyses, Kim et al. demonstrated that EPS from *L. acidophilus* could act as interspecies cell-to-cell signal by downregulating the expression of molecules related to auto-aggregation process and biofilm maintenance in enterohemorrhagic *E. coli* [18]. Furthermore, EPS may exert a competitive inhibition of multivalent carbohydrate–protein interactions [42]. In addition, due to structural similarity between the EPS of lactobacilli and pathogens, beneficial EPS could shield host cell receptors thus reducing pathogens-host cell recognition [27]. The anti-biofilm effects can also be ascribed to the ability of lactobacilli-derived EPS to interfere with the biofilm signaling molecules or to block the glycocalyx receptors at the surface of the pathogens, causing quorum-quenching [40, 43]

Our study highlighted the functional role of *Lactobacillus* EPS in modulating the healthy vaginal microbiota, as we demonstrated that EPS produced by the most preponderant *Lactobacillus* species in the vaginal ecosystem favour the permanence of beneficial bacteria preventing, at the same time, the biofilm formation by opportunistic pathogens. Although experiments carried out in vitro are widely used to screen a wide range of compounds on biofilms, they lack the complexity of in vivo microenvironment, in which an intricate cross-talk between the host and microorganisms harboring the vaginal niche occurs. To date, the capability of orally administered EPS to exert prebiotic properties and antimicrobial activity against gut pathogens has been demonstrated in vivo [44–46], but no similar studies have been carried out for what concern EPS in the vaginal ecosystem. In this regard, further studies are required to confirm in vivo the ability of EPS to modulate microbial biofilms in the vaginal niche, to better elucidate their physiological role and the molecular mechanisms underlying their effects.

Although preliminary, our results support the idea that EPS produced by probiotic bacteria can be employed as a therapeutic/preventive strategy to counteract vaginal infections.

## Materials and methods

### Microorganisms and culture conditions

Lactobacilli included in this study were previously isolated from vaginal swabs of healthy pre-menopause Caucasian women, according to the protocol approved by the Ethics Committee of the University of Bologna, Bologna, Italy (52/2014/U/Tess) [47]. According to a recent reclassification of *Lactobacillus* genus [48], they belong to the species *Lactobacillus crispatus* (BC1, BC3, BC4, and BC5), *Lactobacillus gasseri* (BC9, BC10, BC12, and BC14) and *Limosilactobacillus vaginalis* (BC16 and BC17). Lactobacilli were routinely grown in de Man, Rogosa, and Sharpe broth (MRS) (Difco, Detroit, MI, USA) with the addition of L-cysteine 0.05% (w/v) (Merck, Milan, Italy), at 37 °C; the anaerobic conditions were guaranteed by incubation in anaerobic jars containing Gas-Pak EZ (Beckton, Dickinson and Co., Milan, Italy).

*Escherichia coli* DSM1900, *E. coli* DSM18039 and *Staphylococcus aureus* DSM2569 were purchased from the German Collection of Microorganisms and Cell Cultures GmbH (DSMZ, Braunschweig, Germany). *Staphylococcus lugdunensis* BC102, *Enterococcus faecalis* BC101 and *Enterococcus faecium* BC105 belong to the Department of Pharmacy and Biotechnology of the University of Bologna (Italy). *Staphylococcus aureus* SO88, *Streptococcus agalactiae* SO104, *Candida albicans* SO1 and *Candida glabrata* SO17 were isolated at Sant’Orsola-Malpighi University Hospital of Bologna during routine diagnostic procedures. The microbial identification was obtained by means of a matrix-assisted laser desorption/ionization time-of-flight mass spectrometry (MALDI-TOF MS), using a Bruker Microflex MALDI-TOF MS instrument (Bruker Daltonics) [49]. *Staphylococcus* spp., *E. coli*, *Enterococcus* spp. and *S. agalactiae* were aerobically grown at 37 °C in Brain Heart Infusion medium (BHI) (Difco, Detroit, MI, USA), while *Candida* spp. were aerobically cultured at 35 °C in Sabouraud dextrose medium (SD) (Difco, Detroit, MI, USA).

### Isolation of EPS produced by vaginal lactobacilli

The isolation of EPS from supernatants of *L. crispatus* (BC1, BC4, and BC5) and *L. gasseri* (BC9, BC12, and BC14) was carried out following a protocol reported by Tallon et al. [50], with some modifications. Lactobacilli were initially cultured in 10 mL of MRS for 24 h, and then transferred in 100 mL of fresh medium and grown for a further 24 h, as described above. Afterward, the supernatants of lactobacilli were harvested by centrifugation (2750 ×*g*, 10 min, Centrisart G-16C, Sartorius, Goettingen, Germany) and treated with trichloroacetic acid (Merck, Milan, Italy) at a final concentration of 20% (w/v) for 2 h at 4 °C under mild agitation (100 rpm, Universal Table Shaker 709, ASAL, Milan, Italy). The precipitated proteins were removed through centrifugation (15,000 ×*g*, 10 min, 4 °C) and two volumes of cold absolute ethanol (Merck, Milan, Italy) were added to the supernatants. After an incubation of 18 h at 4 °C, the pellets containing EPS were recovered (6000 ×*g*, 30 min, 4 °C), resuspended in 20 mL of sterile distilled water, and dialyzed against 25 L of demineralized water in a Cellu-Sep^©^ membrane (molecular weight cut-off 6000–8000 Da; Spectra/Por 2 dialysis membrane, Spectrum Laboratories Inc., Rancho Dominguez, CA, USA) for 48 h at room temperature, with three water changes per day. Samples were finally freeze-dried at 0.01 atm and −  47 °C (Christ Freeze Dryer ALPHA 1–2, Milan, Italy). Lyophilized EPS from *L. crispatus* (EPS-BC1, EPS-BC4, EPS-BC5) and *L. gasseri* (EPS-BC9, EPS-BC12, EPS-BC14) strains were stored at 4 °C until their use.

### Determination of moisture content

Weighted amounts of lyophilized EPS samples were submitted to a three-step drying process using a Thermomixer (Eppendorf comfort) as follows: 80 °C for 30 h, 90 °C for the subsequent 15 h and 99 °C for 5 h. At the end of each step, samples were cooled down and weighted and the amount of weight loss was determined. Used temperatures are largely below the reported degradation temperature of EPS, i.e. 230 °C [24]. In agreement, visual inspection of samples did not show any significant change.

### Determination of monosaccharide composition of EPS

EPS were analyzed through LC-UV and LC–MS analyses to determine the monosaccharide composition. All reagents employed were purchased from Merck; the organic solvents were of HPLC grade. LC-UV and LC–MS analyses were performed on a Kromasil C18 (4.6 × 150 mm; 5 µm; 100°A; Phenomenex, Torrance, CA, USA) chromatographic column. Instrumental set-ups are detailed in subsequent sections.

### Preparation of samples

For the analyses, EPS underwent acid hydrolysis followed by derivatization. Lyophilized EPS (1 mg) were solubilized in 250 μL of HCl 4 M and incubated at 99 °C for 2 h under gentle shaking (300 rpm, Thermomixer Comfort; Eppendorf, Milan, Italy) for hydrolysis. 250 μL of NaOH 4 M were then added to the samples for neutralization.

Derivatization of monosaccharides was carried out slightly modifying a previously reported procedure [25]. In details, 120 μL of the hydrolysed solutions were mixed with 180 μL of NaOH 0.5 M. 200 μL of the resulting samples were mixed with 200 μL of PMP (0.5 M in methanol) and incubated at 70 °C for 1 h under gentle shaking (300 rpm). After cooling at room temperature, 200 μL of HCl 0.3 M and 300 μL of Tris buffer (1.5 M, pH 7.00) were subsequentially added for neutralization. The resulting mixtures were extracted 3 times with 500 μL of dichloromethane to remove the excess of PMP. Samples were aliquoted and stored at − 20 °C until analysis. Standard solutions (6.25 mM) of d-mannose, d-glucosamine, d-galactosamine, d-rhamnose, d-glucose, d-galactose,d-xylose, and d-fucose were derivatized following the same procedure.

The whole procedure was performed in triplicate for each sample.

### LC-UV analysis

The LC-UV analyses were performed slightly modifying the method proposed by Wang et al. [51] on a Jasco HPLC system (Jasco PU-2080 Plus equipped with detector UV-2070 Plus, Pfungstadt, Germany) equipped with an autosampler (Jasco AS-2055 Plus) and a column oven (Jasco CO-2067 Plus) and using a C18 column (Kromasil; 4,6 × 150 mm; 5 µm; 100°A; Phenomenex, Torrance, CA, USA) termostated at 20 °C. A gradient elution was developed with the mobile phase A (sodium acetate buffer, 100 mM, pH 4.00) and B (acetonitrile). Mobile phase B was increased from 17.0% to 18.5% in 10 min and from 18.5% to 25.0% in following 20 min. The column was equilibrated with the starting condition for 6 min before the next injection. Flow rate was set at 1.2 mL/min and the injection volume was 20 μL. UV detection was performed at 254 nm.

To build calibration curves, the 1.1 mM solution of each derivatized monosaccharides was diluted with sodium acetate buffer 100 mM pH 4.00 to get working solutions ranging from 0.098 to 25 μM for d-Mannose, from 0.098 to 50 μM for d-Glucosamine, from 0.39 to 25 μM for d-Galactosamine and d-Fucose, from 0.20 to 25 μM for d-Rhamnose and d-Galactose, from 0.20 to 50 μM for d-Glucose and 0.098 to 50 μM for -Xylose. Standard solutions were analysed by liquid chromatography-UV (LC–UV) method reported below. Limit of quantitation (LOQ) values were determined by performing LC-UV analysis on incremental dilutions of standard solutions and applying the formula (Eq. 1):

1 $${\text{LOQ}} = {1}0\left( {\sigma {\text{b}}/{\text{a}}} \right)$$

where “a” is the slope and “σb” is the standard deviation of the y-intercept of the regression curves [52].

For the quantitation of all monosaccharides except d-Xylose, derivatized EPS were diluted 1:27 with sodium acetate buffer 100 mM, pH 4.00; for quantifying d-Xylose samples were diluted 1:10.

### LC–MS analysis

The LC–MS analyses were performed on a UPLC system (Acquity, quaternary Solvent Menager, Manchester, UK) coupled in line with a Q-TOF mass spectrometer (Xevo G2-XS QTof, Waters, Manchester, UK) equipped with an ESI interface operating in positive ion mode. A C18 column (Kromasil; 4,6 × 150 mm; 5 µm; 100°A; Phenomenex, Torrance, CA, USA) thermostated at 30 °C was used for the analyses. A gradient elution was developed with mobile phase A (ammonium acetate buffer, 100 mM, pH 5.51) and B (acetonitrile): mobile phase B was increased from 18.0% to 19.2% in 8 min, from 19.2% to 23.0% in the following 4 min and from 23.0% to 26.0 in the last 3 min. The column was equilibrated with starting conditions for 8 min before the next injection. Flow rate was set at 1.0 mL/min and injection volume at 10 μL.

Mass spectrometer operated in high sensitivity mode using a capillary voltage of 3.0 kV and a cone voltage of 0.8 kV. Cone and desolvation gas flow were 50 and 1200 L/h, respectively, while the source and desolvation gas temperature were 150 and 600 °C, respectively. Leucine enkephalin (0.1 ng/μL) was used as lock mass (*m/z* 556.2771). Data were acquired in MS^E^ mode from *m/z* 50 to 1200, creating two discrete and independent interleaved acquisition functions. The first acquisition, set at 6.0 eV of collision energy, collects low energy of unfragmented data, while the second has a collision energy ramp from 20 to 30 eV and collects fragmented data. Argon was used for collision-induced dissociation. Unifi software (Waters, Manchester, UK) was used for visualization and alignment of low- and high-energy information.

### Effects of EPS on biofilms of lactobacilli and opportunistic pathogens

EPS-BC1, EPS-BC4, EPS-BC5, EPS-BC9, EPS-BC12, and EPS-BC14 were sought for the effects on the biofilms of lactobacilli belonging to the species *L. crispatus* (BC1, BC3, BC4, and BC5), *L. gasseri* (BC9, BC10, BC12, and BC14) and *L. vaginalis* (BC16 and BC17). The impact of EPS on biofilms of opportunistic pathogens (*E. coli* DSM1900, *E. coli* DSM2569, *S. aureus* DSM2569, *S. aureus* SO88, *S. lugdunensis* BC102, *E. faecalis* BC101, *E. faecium* BC105, *S. agalactiae* SO104, *C. albicans* SO1, and *C. glabrata* SO17) were also investigated.

In all cases, the formation of biofilms was evaluated by two methods, namely crystal violet staining and MTT assay. For each experiment, three different batches of EPS were analyzed in triplicate on three independent assays.

### Biofilm formation

Lyophilized EPS as moisturized samples were solubilized in sterile distilled water at concentrations of 0.2 mg/mL, 1 mg/mL and 2 mg/mL. Microorganisms were subcultured twice as previously reported, and then diluted in the appropriate growth medium to obtain a final cell concentration of 10^7^ CFU/mL. Suspensions of aerobic opportunistic pathogens (100 μL) were inoculated in 96 multi-well flat-bottom plates (Corning Inc., Pisa, Italy) [53, 54], while the suspensions of lactobacilli (100 μL) were used to inoculate 96 multi-well round-bottom plates (Corning Inc., Pisa, Italy) [55] which favour the growth of microaerophilic bacteria. 100 μL of EPS solutions were added to the wells, to test final concentrations of 0.1 mg/mL, 0.5 mg/mL, and 1 mg/mL. Control wells contained 100 μL of microbial suspension and 100 μL of distilled water. Wells containing medium (MRS, BHI, or SD) and distilled water served as blank, while wells filled with medium and EPS solutions were included as sterility controls. Two microplates for each microorganism were set up and incubated under anaerobic (lactobacilli) [55] or aerobic conditions (opportunistic pathogens) [53, 54] for 72 h to allow biofilm formation.

For the analysis on mixed biofilms, mixed microbial suspensions were prepared in the appropriate growth medium as follows: (i) *L. crispatus* BC4 + *L. crispatus* BC5; (ii) *L. gasseri* BC10 + *L. gasseri* BC12; (iii) *E. coli* DSM1900 + *S. aureus* DSM2569; (iv) *C. albicans* SO1 + *C. glabrata* SO17). The concentration of each microorganism in mixed suspensions was 5 × 10^6^ CFU/mL, so that the final microbial concentration was 10^7^ CFU/mL. EPS solutions were tested at final concentration of 1 mg/mL and the biofilm formation was carried out for 72 h as described above.

### Biofilm quantification by crystal violet staining

The first microplate was used for biofilm biomass quantification by crystal violet staining following protocols already reported for lactobacilli [55], other bacteria [53] and *Candida* spp. [54], with slight modifications. Culture supernatants were discarded, adherent microorganisms were gently washed twice with sterile saline (NaCl 0.9%, w/v, Merck) and fixed with 200 μL of absolute ethanol. Lactobacilli biofilms were stained with 0.1% (w/v) of crystal violet (CV, Merck) for 25 min, biofilms of *E. coli*, *S. agalactiae*, *Staphylococcus* spp. and *Enterococcus* spp. were stained with 0.41% CV (w/v) for 10 min, and 1% CV (w/v) was used to stain biofilms of *Candida* spp. for 5 min. The excess of dye was removed by washing wells three times with sterile saline and microplates were dried overnight at room temperature. The dye bound to adherent cells was resolubilized in 200 μL of ethanol and the absorbance (ABS) was measured at 595 nm by a microplate reader (EnSpire Multimode Plate Reader, PerkinElmer Inc., Waltham, MA, USA). The formation of biofilms in the presence of EPS was expressed in percentage relative to the control wells, as follows (Eq. 2):

2 $${\text{Formation of biofilm }}\left( \% \right) = ({{\text{ABS biofilm with EPS}} \mathord{\left/ {\vphantom {{\text{ABS biofilm with EPS}} {{\text{ABS}}_{{}} {\text{control biofilm}}}}} \right. \kern-0pt} {{\text{ABS}}_{{}} {\text{control biofilm}}}}) \times 100$$

### Biofilm quantification by MTT assay

The second plate was used for the quantification of the biofilm metabolic activity by MTT assay. A stock solution at 5 mg/mL of 3-(4,5-dimethyl-2-thiazolyl)-2,5-diphenyl-2H-tetrazolium bromide (MTT, Merck) was prepared in distilled water. After removal of supernatants, the adherent biofilms were incubated in dark with 100 μL of MTT diluted 1/10 in culture medium (MRS, BHI or SD) at 37 °C for 3 h (bacteria) [39] or at 35 °C for 4 h (fungi) [56]. Afterwards, the medium was removed from wells and replaced with 200 μL of isopropanol (Merck) to solubilize the formazan crystals formed, which were quantified by reading ABS at 570 nm. Results were expressed in percentages compared to the control wells, following the Eq. 2.

### Statistical analysis

All analyses were performed at least in duplicate on three independent experiments (*n* = 3) and results were expressed as mean ± standard deviation (SD). The significance of the differences in the monosaccharide compositions was assessed by applying unpaired t-test. One-way ANOVA followed by Tukey’s correction for multiple comparison and Wilcoxon paired-rank test were used to analyse biofilm data. All statistical analyses were performed using GraphPad Prims version 9.4.1 for Windows (GraphPad Software, San Diego, CA, USA, www.graphpad.com). Differences were deemed significant for *p* < 0.05.

## Supplementary Information

**Additional file 1: Table S1.** Retention time, regression analysis, and LOD for the PMP-monosaccharides found in EPS. **Figure S1.** Representative chromatographic analysis of (**A**) derivatized monosaccharides released upon hydrolysis of EPS-BC5, and (**B**) mixture of eight standard PMP-monosaccharides. Peaks: 1: d-mannose; 2: d-glucosamine; 3: d-rhamnose; 4: d-galactosamine; 5: d-glucose; 6:d-galactose; 7: d-xylose; 8:d- fucose. Exceeding PMP elutes with the shortest retention time. **Figure. S2.** Representative LC-MS chromatogram of PMP-monosaccharides released upon hydrolysis of EPS-BC5. The peak identification was achieved on the basis of analyte retention time, molecular weight of the parent ions, and fragment ions (Unifi Software). **Figure. S3.** Effects of (**A**) EPS-BC1, (**B**) EPS-BC4, (**C**) EPS-BC5, (**d**) EPS-BC9, (**E**) EPS-BC12 and (**F**) EPS-BC14 at three concentrations (0.1, 0.5, 1 mg/mL) on the biofilms of lactobacilli, evaluated through CV staining. The results are expressed in percentage with respect to control (100%, red line) (mean ± SD, *n *= 3). *p < 0.05. **Figure. S4.** Effects of (**A**) EPS-BC1, (**B**) EPS-BC4, (**C**) EPS-BC5, (**D**) EPS-BC9, (**E**) EPS-BC12 and (**F**) EPS-BC14 at three concentrations (0.1, 0.5, 1 mg/mL) on the biofilms of lactobacilli, evaluated through MTT assay. The results are expressed in percentage with respect to control (100%, red line) (mean ± SD, *n *= 3). *p < 0.05. **Figure. S5.** Effects of (**A**) EPS-BC1, (**B**) EPS-BC4, (**C**) EPS-BC5, (**D**) EPS-BC9, (**E**) EPS-BC12 and (**F**) EPS-BC14 at three concentrations (0.1, 0.5, 1 mg/mL) on the biofilms of pathogens, evaluated through CV staining. The results are expressed in percentage with respect to control (100%, red line) (mean ± SD, *n *= 3). *p < 0.05. **Figure. S6.** Effects of (**A**) EPS-BC1, (**B**) EPS-BC4, (**C**) EPS-BC5, (**D**) EPS-BC9, (**E**) EPS-BC12 and (**F**) EPS-BC14 at three concentrations (0.1, 0.5, 1 mg/mL) on the biofilms of pathogens, evaluated through MTT assay. The results are expressed in percentage with respect to control (100%, red line) (mean ± SD, *n *= 3). *p < 0.05. **Figure. S7.** Correlation graphics between results collected through CV staining and MTT assay towards biofilms of lactobacilli (**A**) and pathogens (**B**). Data obtained testing EPS (EPS-BC1, EPS-BC4, EPS-BC5, EPS-BC9, EPS-BC12 and EPS-BC14) at three concentrations (0.1, 0.5 and 1 mg/mL) are included in the analyses.

## Data Availability

All data generated or analyzed during this study are included in this published article [and its additional files].
