# Supplementary material for: Exopolysaccharides from vaginal lactobacilli modulate microbial biofilms
Source: Microb Cell Fact. 2023 Mar 8;22:45. doi: 10.1186/s12934-023-02053-x (PMC9993704; doi:10.1186/s12934-023-02053-x)
Supplement: Supplementary file 1 — Additional file 1: Table S1. Retention time, regression analysis, and LOD for the PMP-monosaccharides found in EPS. Figure S1. Representative chromatographic analysis of (A) derivatized monosaccharides released upon hydrolysis of EPS-BC5, and (B) mixture of eight standard PMP-monosaccharides. Peaks: 1: d-mannose; 2: d-glucosamine; 3: d-rhamnose; 4: d-galactosamine; 5: d-glucose; 6:d-galactose; 7: d-xylose; 8:d- fucose. Exceeding PMP elutes with the shortest retention time. Figure. S2. Representative LC-MS chromatogram of PMP-monosaccharides released upon hydrolysis of EPS-BC5. The peak identification was achieved on the basis of analyte retention time, molecular weight of the parent ions, and fragment ions (Unifi Software). Figure. S3. Effects of (A) EPS-BC1, (B) EPS-BC4, (C) EPS-BC5, (d) EPS-BC9, (E) EPS-BC12 and (F) EPS-BC14 at three concentrations (0.1, 0.5, 1 mg/mL) on the biofilms of lactobacilli, evaluated through CV staining. The results are expressed in percentage with respect to control (100%, red line) (mean ± SD, n = 3). *p < 0.05. Figure. S4. Effects of (A) EPS-BC1, (B) EPS-BC4, (C) EPS-BC5, (D) EPS-BC9, (E) EPS-BC12 and (F) EPS-BC14 at three concentrations (0.1, 0.5, 1 mg/mL) on the biofilms of lactobacilli, evaluated through MTT assay. The results are expressed in percentage with respect to control (100%, red line) (mean ± SD, n = 3). *p < 0.05. Figure. S5. Effects of (A) EPS-BC1, (B) EPS-BC4, (C) EPS-BC5, (D) EPS-BC9, (E) EPS-BC12 and (F) EPS-BC14 at three concentrations (0.1, 0.5, 1 mg/mL) on the biofilms of pathogens, evaluated through CV staining. The results are expressed in percentage with respect to control (100%, red line) (mean ± SD, n = 3). *p < 0.05. Figure. S6. Effects of (A) EPS-BC1, (B) EPS-BC4, (C) EPS-BC5, (D) EPS-BC9, (E) EPS-BC12 and (F) EPS-BC14 at three concentrations (0.1, 0.5, 1 mg/mL) on the biofilms of pathogens, evaluated through MTT assay. The results are expressed in percentage with respect to control (100%, red line) (mean ± [file 12934_2023_2053_MOESM1_ESM.pdf]

# Exopolysaccharides from vaginal lactobacilli modulate microbial biofilms

Barbara Giordani[1], Marina Naldi[1], Vanessa Croatti[1], Carola Parolin[1], Ülfet Erdoğan[2], Manuela Bartolini[1], Beatrice Vitali[1*]

[1] Department of Pharmacy and Biotechnology, University of Bologna, Bologna, Italy

[2] Yeditepe University, Istanbul, Turkey

[*] Corresponding to: b.vitali@unibo.it

## Additional file

**Index**

**Table S1**: Retention time, regression analysis, and LOD for the PMP-monosaccharides found in EPS………………. **p2**

**Figure S1**: Representative chromatographic analysis of (**A**) derivatized monosaccharides released upon hydrolysis of EPS-BC5, and (**B**) mixture of eight standard PMP-monosaccharides. Peaks: 1: D-mannose; 2: D-glucosamine; 3: D-rhamnose; 4: D-galactosamine; 5: D-glucose; 6: D-galactose; 7: D-xylose; 8: D- fucose. Exceeding PMP elutes with the shortest retention time………………………………………………………........................................ **p3**

**Fig. S2** Representative LC-MS chromatogram of PMP-monosaccharides released upon hydrolysis of EPS-BC5. The peak identification was achieved on the basis of analyte retention time, molecular weight of the parent ions, and fragment ions (Unifi Software)…………………………………………………………………….......................... **p4**

**Fig. S3** Effects of (**A**) EPS-BC1, (**B**) EPS-BC4, (**C**) EPS-BC5, (**D**) EPS-BC9, (**E**) EPS-BC12 and (**F**) EPS-BC14 at three concentrations (0.1, 0.5, 1 mg/mL) on the biofilms of lactobacilli, evaluated through CV staining. The results are expressed in percentage with respect to control (100%, red line) (mean ± SD, $n = 3$). * $p < 0.05$……………………………………………………………………………………………………………**p5**

**Fig. S4** Effects of (**A**) EPS-BC1, (**B**) EPS-BC4, (**C**) EPS-BC5, (**D**) EPS-BC9, (**E**) EPS-BC12 and (**F**) EPS-BC14 at three concentrations (0.1, 0.5, 1 mg/mL) on the biofilms of lactobacilli, evaluated through MTT assay. The results are expressed in percentage with respect to control (100%, red line) (mean ± SD, $n = 3$). * $p < 0.05$............................ **p6**

**Fig. S5** Effects of (**A**) EPS-BC1, (**B**) EPS-BC4, (**C**) EPS-BC5, (**D**) EPS-BC9, (**E**) EPS-BC12 and (**F**) EPS-BC14 at three concentrations (0.1, 0.5, 1 mg/mL) on the biofilms of pathogens, evaluated through CV staining. The results are expressed in percentage with respect to control (100%, red line) (mean ± SD, $n = 3$). * $p < 0.05$…………………. **p7**

**Fig. S6** Effects of (**A**) EPS-BC1, (**B**) EPS-BC4, (**C**) EPS-BC5, (**D**) EPS-BC9, (**E**) EPS-BC12 and (**F**) EPS-BC14 at three concentrations (0.1, 0.5, 1 mg/mL) on the biofilms of pathogens, evaluated through MTT assay. The results are expressed in percentage with respect to control (100%, red line) (mean ± SD, $n = 3$). * $p < 0.05$……………………... **p8**

**Fig. S7** Correlation graphics between results collected through CV staining and MTT assay towards biofilms of lactobacilli (**A**) and pathogens (**B**). Data obtained testing EPS (EPS-BC1, EPS-BC4, EPS-BC5, EPS-BC9, EPS-BC12 and EPS-BC14) at three concentrations (0.1, 0.5 and 1 mg/mL) are included in the analyses………………….. **p9**

**Table S1** Retention time, regression analysis, and LOD for the PMP-monosaccharides found in EPS.

| Monosaccharide | Retention time (min) | Linearity range (μM) | Regression equation, y = mx + q | | Correlation coefficient | Limit of quantification (LOQ) (μM) |
|---|---|---|---|---|---|---|
| | | | m | q | | |
| D-mannose | 9.8 | 0.098-25 | 20932 | -945 | 0.9999 | 0.064 |
| D-glucosamine | 11.4 | 0.20-50 | 19487 | -1604 | 0.9999 | 0.10 |
| D-rhamnose | 13.4 | 0.39-25 | 18047 | -1347 | 0.9997 | 0.34 |
| D-galactosamine | 16.4 | 0.20-50 | 23338 | -388 | 0.9997 | 0.15 |
| D-glucose | 22.3 | 0.20-50 | 14146 | -1587 | 0.9996 | 0.15 |
| D-galactose | 23.4 | 0.20-25 | 13045 | -461 | 0.9994 | 0.18 |
| D-xylose | 25.1 | 0.098-50 | 15293 | 981 | 0.9994 | 0.064 |
| D-fucose | 27.2 | 0.39-25 | 13834 | -732 | 0.9991 | 0.20 |

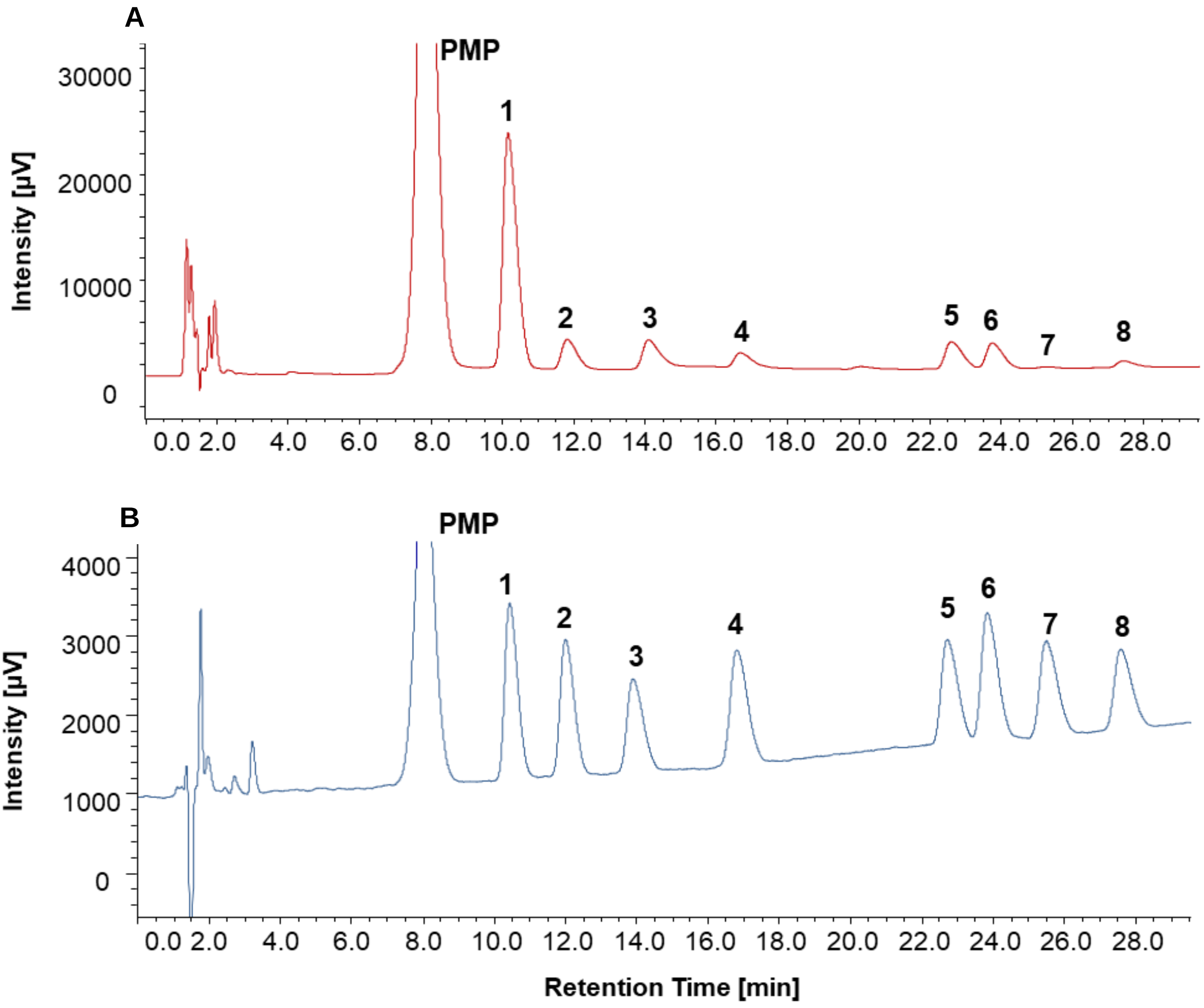


**Fig. S1** Representative chromatographic analysis of (**A**) derivatized monosaccharides released upon hydrolysis of EPS-BC5, and (**B**) mixture of eight standard PMP-monosaccharides. Peaks: 1: D-mannose; 2: D-glucosamine; 3: D-rhamnose; 4: D-galactosamine; 5: D-glucose; 6: D-galactose; 7: D-xylose; 8: D- fucose. Exceeding PMP elutes with the shortest retention time.

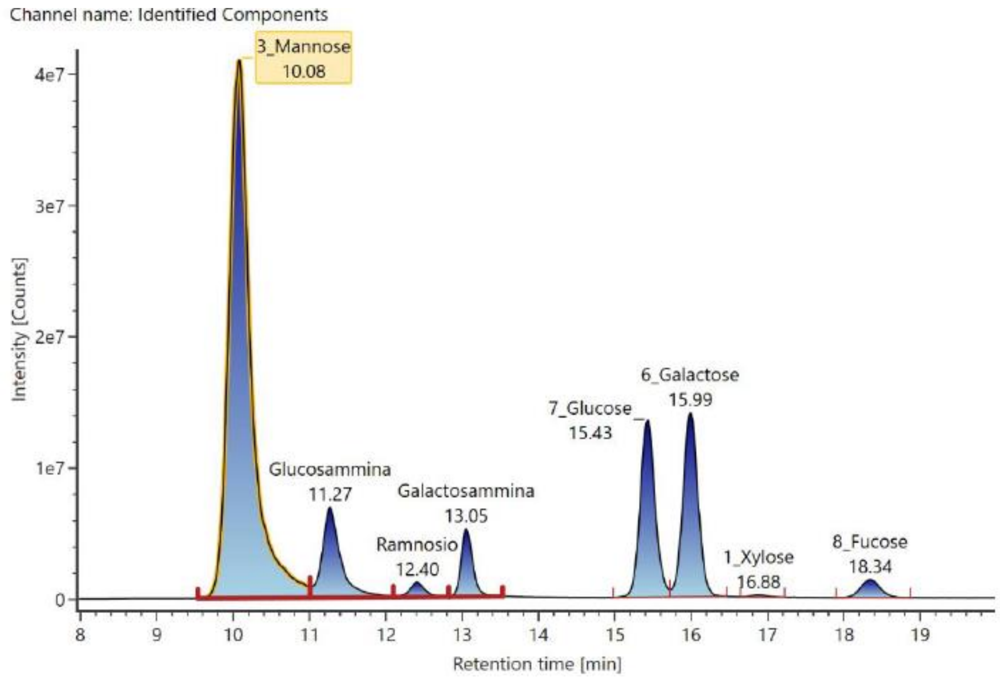


**Fig. S2** Representative LC-MS chromatogram of PMP-monosaccharides released upon hydrolysis of EPS-BC5. The peak identification was achieved on the basis of analyte retention time, molecular weight of the parent ions, and fragment ions (Unifi Software).

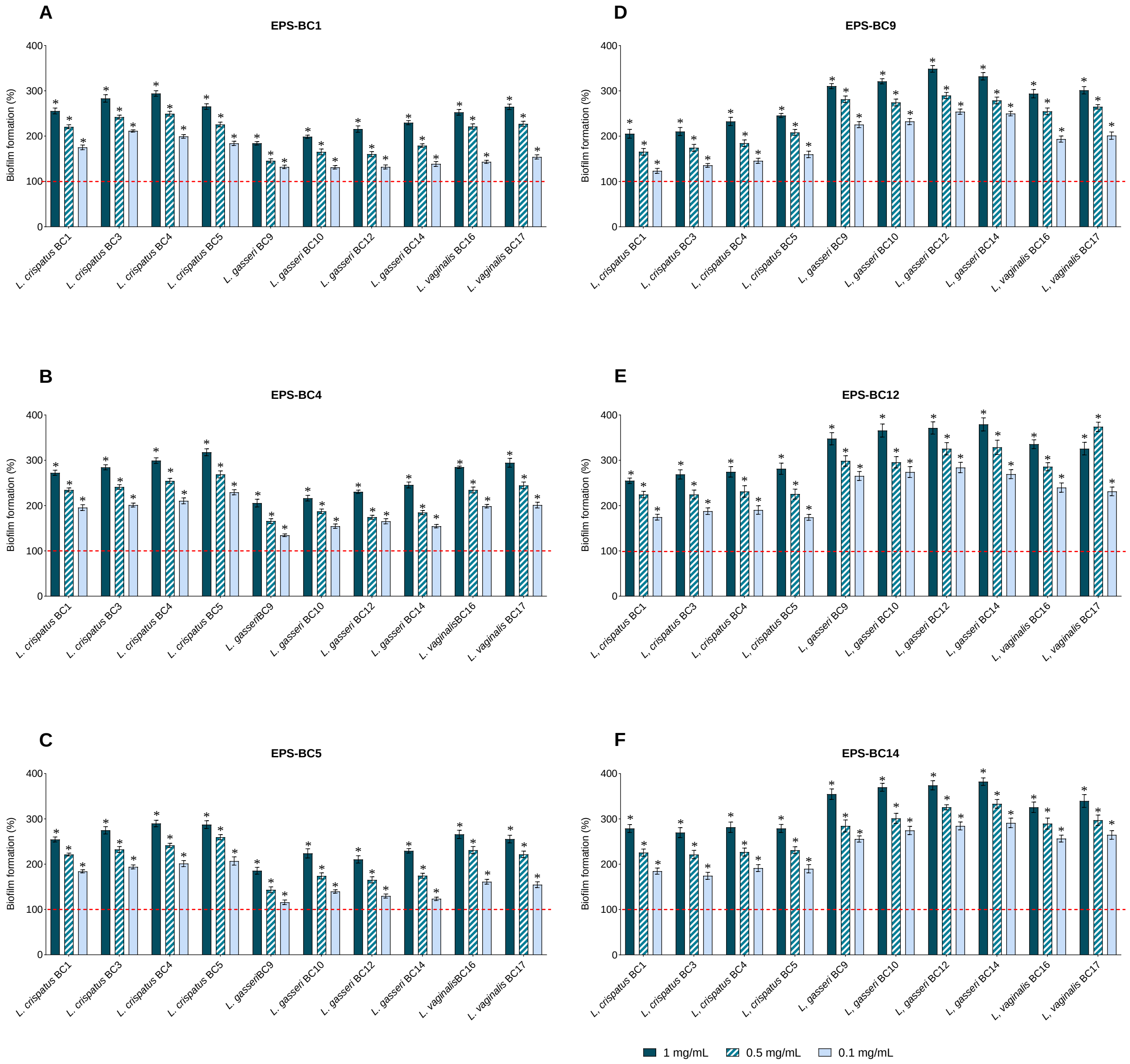


**Fig. S3** Effects of (**A)** EPS-BC1, (**B**) EPS-BC4, (**C**) EPS-BC5, (**D**) EPS-BC9, (**E**) EPS-BC12 and (**F**) EPS-BC14 at three concentrations (0.1, 0.5, 1 mg/mL) on the biofilms of lactobacilli, evaluated through CV staining. The results are expressed in percentage with respect to control (100%, red line) (mean ± SD, $n$ = 3). * $p < 0.05$.

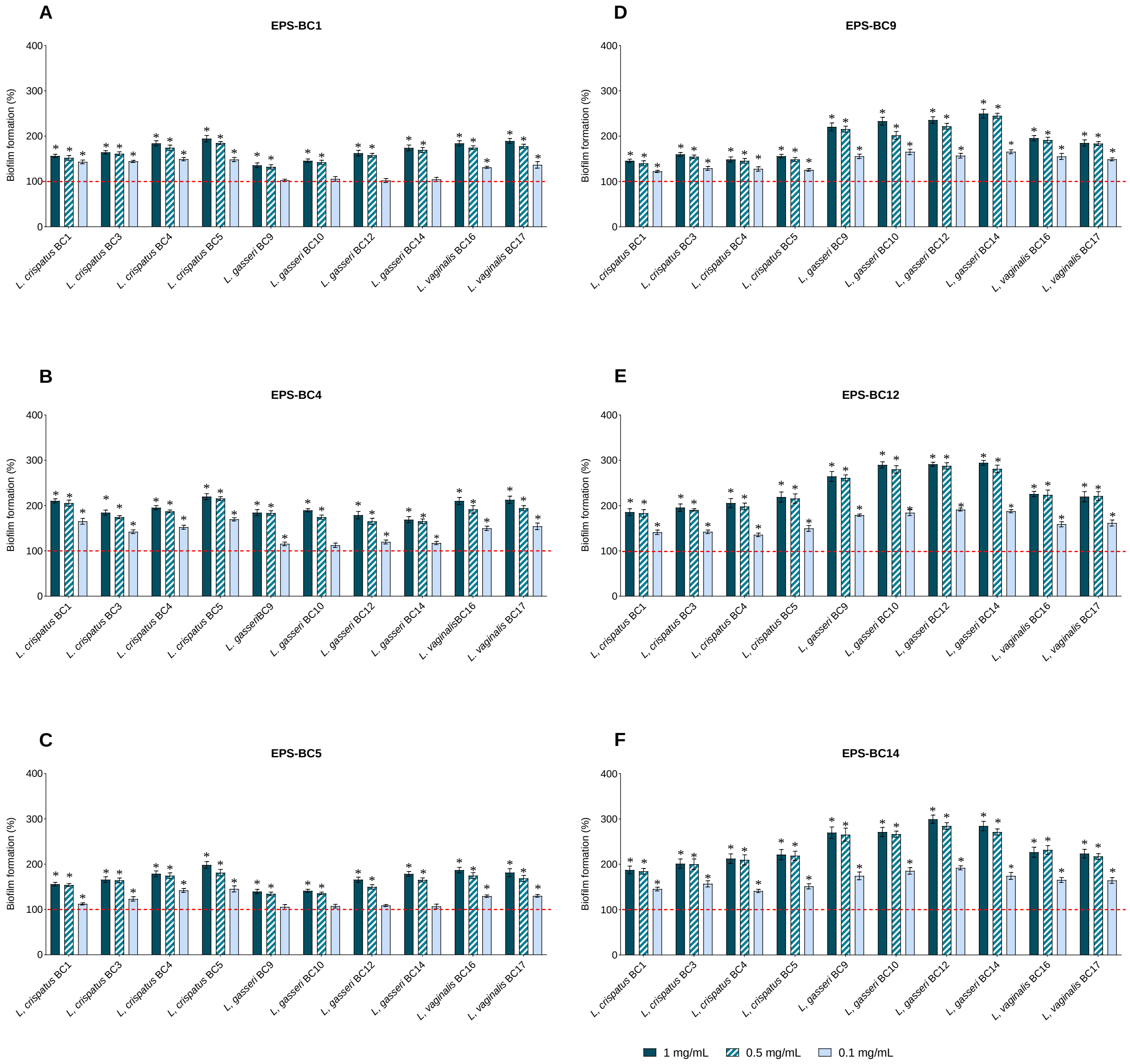


**Fig. S4** Effects of (**A**) EPS-BC1, (**B**) EPS-BC4, (**C**) EPS-BC5, (**D**) EPS-BC9, (**E**) EPS-BC12 and (**F**) EPS-BC14 at three concentrations (0.1, 0.5, 1 mg/mL) on the biofilms of lactobacilli, evaluated through MTT assay. The results are expressed in percentage with respect to control (100%, red line) (mean ± SD, $n = 3$). * $p < 0.05$.

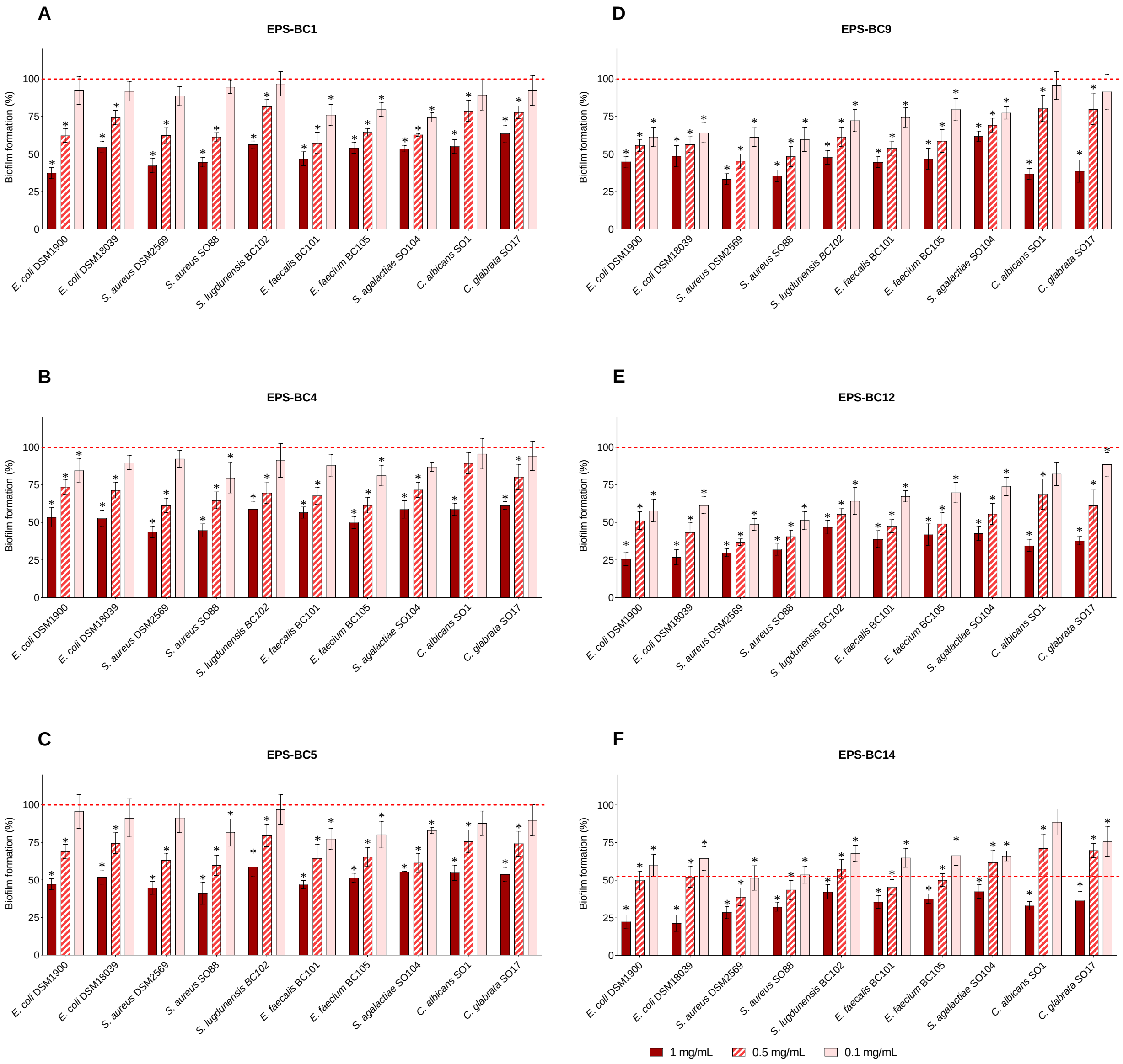


**Fig. S5** Effects of (**A**) EPS-BC1, (**B**) EPS-BC4, (**C**) EPS-BC5, (**D**) EPS-BC9, (**E**) EPS-BC12 and (**F**) EPS-BC14 at three concentrations (0.1, 0.5, 1 mg/mL) on the biofilms of pathogens, evaluated through CV staining. The results are expressed in percentage with respect to control (100%, red line) (mean ± SD, $n = 3$). * $p < 0.05$.

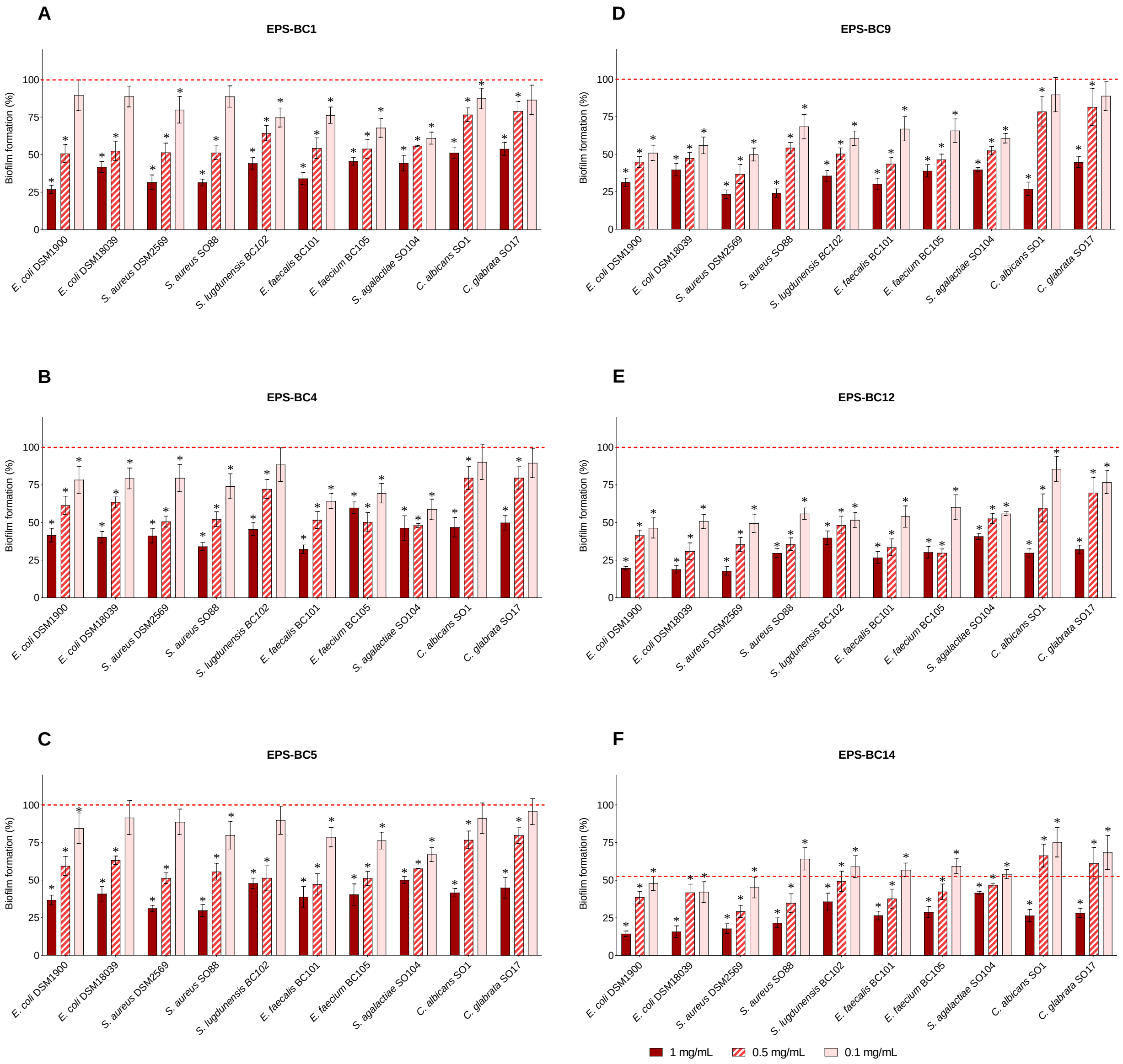


**Fig. S6** Effects of (**A**) EPS-BC1, (**B**) EPS-BC4, (**C**) EPS-BC5, (**D**) EPS-BC9, (**E**) EPS-BC12 and (**F**) EPS-BC14 at three concentrations (0.1, 0.5, 1 mg/mL) on the biofilms of pathogens, evaluated through MTT assay. The results are expressed in percentage with respect to control (100%, red line) (mean ± SD, $n = 3$). * $p < 0.05$.

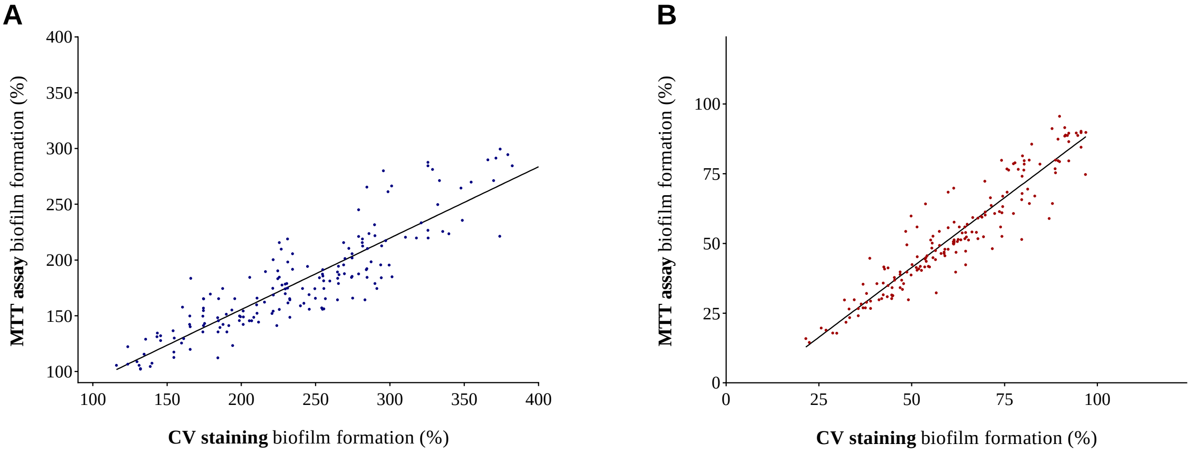


**Fig. S7** Correlation graphics between results collected through CV staining and MTT assay towards biofilms of lactobacilli (**A**) and pathogens (**B**). Data obtained testing EPS (EPS-BC1, EPS-BC4, EPS-BC5, EPS-BC9, EPS-BC12 and EPS-BC14) at three concentrations (0.1, 0.5 and 1 mg/mL) are included in the analyses.
